# A Mathematical Model of Salivary Gland Duct Cells

**DOI:** 10.1007/s11538-022-01041-3

**Published:** 2022-07-07

**Authors:** Shan Su, John Rugis, Amanda Wahl, Sam Doak, Yating Li, Vinod Suresh, David Yule, James Sneyd

**Affiliations:** 1 Department of Mathematics, University of Auckland, 38 Princess Street, Auckland, 1010 New Zealand; 2 Auckland Biomedical Engineering Institute, University of Auckland, 70 Symonds Street, Auckland, 1010 New Zealand; 3 Department of Engineering Science, University of Auckland, 70 Symonds Street, Auckland, 1010 New Zealand; 4 School of Medicine and Dentistry, University of Rochester, 601 Elmwood Ave, Rochester, NY 14642 USA

**Keywords:** Salivary gland, Ion transporters, 3D reconstruction, Immunostaining, Mathematical modelling

## Abstract

Saliva is produced in two stages in the salivary glands: the secretion of primary saliva by the acinus and the modification of saliva composition to final saliva by the intercalated and striated ducts. In order to understand the saliva modification process, we develop a mathematical model for the salivary gland duct. The model utilises the realistic 3D structure of the duct reconstructed from an image stack of gland tissue. Immunostaining results show that TMEM16A and aquaporin are expressed in the intercalated duct cells and that ENaC is not. Based on this, the model predicts that the intercalated duct does not absorb Na$^+$ and Cl$^-$ like the striated duct but secretes a small amount of water instead. The input to the duct model is the time-dependent primary saliva generated by an acinar cell model. Our duct model produces final saliva output that agrees with the experimental measurements at various stimulation levels. It also shows realistic biological features such as duct cell volume, cellular concentrations and membrane potentials. Simplification of the model by omission of all detailed 3D structures of the duct makes a negligible difference to the final saliva output. This shows that saliva production is not sensitive to structural variation of the duct.

## Introduction

Saliva is a fluid mixture of electrolytes, protein and chemical compounds, which lubricates and protects the oral mucosa and facilitates mastication and swallowing. The condition of producing insufficient saliva is called xerostomia, which may lead to severe consequences such as oral pain, mouth infections, dental caries and loss of teeth (Daniels and Wu 2000). Xerostomia is a common symptom of Sjögren’s syndrome, one of the most common autoimmune diseases (Vivino et al. 2019). Therefore, it is important to understand the mechanism of saliva production for disease treatment. In this paper, we aim to mathematically model the functionality of salivary glands.

Mammals have three major salivary glands, the parotid gland, the submandibular gland and the sublingual gland. In these glands, most of the salivary fluid is produced by the secretory end pieces called the acini. The acinus produces a primary saliva that has high Na$^+$ and Cl$^-$ concentrations and a low K$^+$ concentration. The primary saliva flows down a duct network which extracts much of the Na$^+$ and Cl$^-$ and secretes some K$^+$, reducing the saliva osmolarity (Nauntofte 1992; Melvin et al. 2005). This process converts the primary saliva to the final saliva that flows into the mouth.

Each rat acinus is thought to comprise of 6–8 acinar cells (Tamarin and Sreebny 1965), whereas our Keyence microscope imaged mouse submandibular gland stacks show on average 12–14 acinar cells per acinus (Appendix ). Each acinar cell is a polarised epithelial cell, with an apical end that faces the saliva-collecting lumen of the acinus, and a basal side that faces the interstitial fluid. Neuronal stimulation induces calcium oscillations inside an acinar cell, which leads to the activation of ion channels, transepithelial ion movement and consequent primary saliva secretion. Primary saliva has a Na$^+$ concentration of around 140 mM, a Cl$^-$ concentration of 120 mM and a low K$^+$ concentration of 10 mM.

The salivary gland duct has four principal sections. In the order that saliva flows through, these are the intercalated duct (ID), the striated duct (SD), the excretory duct (ED) and the main excretory ducts (Amano et al. 2012). The classical view of the salivary duct is that the ID and SD absorb Na$^+$ and Cl$^-$ and secrete K$^+$, resulting in a final saliva that has a low Na$^+$ and Cl$^-$ concentrations and high K$^+$ concentration. The exact final saliva composition varies depending on the animal, gland and stimulation type, but is always hypoosmotic relative to the interstitial fluid. The complete duct is thought to be water-impermeable on the luminal side to prevent water from leaving the lumen following the osmolarity gradient (Ohana 2015). The ED and the main excretory ducts transport the saliva without altering its composition. As our focus is ion transport along the duct, we limit our study to ID and SD.

In all animal cells, the phospholipid bilayer cell membrane is impermeable to water, polar molecules and macromolecules; ions and water move through transport proteins embedded on the cell membrane (Lodish 2016). Like the acinar cells, the salivary gland duct cells are also polarised epithelial cells with apical and basolateral membranes. The cells form an integrated layer that lines the duct. The type and densities of the transport proteins to each side of the duct cells determine the duct functionalities.

The SD cells absorb Na$^+$ through apical ENaC channels (Catalán et al. 2010) and the intracellular Na$^+$ are effectively removed via strong expression of basolateral NaK ATPase, as indicated in our immunostaining results and in Winston et al. (1988). Apical Maxi-K channels (Catalán et al. 2014; Nakamoto et al. 2008) secrete K$^+$ into the lumen and basolateral K$^+$ channels (Zhou et al. 2010; Liu et al. 1999) maintain a physiological membrane potential. We show using our model that the transportation of Cl$^-$ and HCO$_3^-$ is achieved through the orchestrated work of CFTR channels (Catalán et al. 2010), Na$^+$/H$^+$ exchangers (Park et al. 1999; Zhao et al. 1995; He et al. 1996), Na$^+$/HCO$_3^-$ cotransporters (Luo et al. 2001; Li et al. 2006) and anion exchangers (Zhao et al. 1995).

The classical view of the ID is that it transports ions similarly to the SD. In this paper, we argue against that view based on some experimental evidence. We show that ID cells express TMEM16A (a Calcium activated Chloride Channel, or CaCC), AQP5 and almost no ENaC, which indicates that ID cells are not Na$^+$ and Cl$^-$ absorbent and are water secretive. Gao et al. (2011) has shown that saliva secretion is enhanced in radiation damaged parotid gland through the addition of aquaporins, which agrees with the water secretory nature of the ID cells.

There have been some models of the salivary gland ducts. Patterson et al. (2012) developed a duct model for the mouse submandibular gland, which does not track the cellular pH and the corresponding ion transporters. Fong et al. (2017) developed a duct model fitted to a minipig parotid gland. Both models assume straight tubular duct geometries, and thus, it is not clear whether the intricate branching structure of the duct matters to saliva production. Neither of the models consider the dynamic response of a duct to a realistic time-varying input of primary saliva. They also both lack the experimental evidence on the spatial variation in ion transporter expression to inform the differences between ID and SD.

We develop a model of the mouse salivary gland that includes several acini and the ID and SD extending from them, where the duct structure is based on the 3D reconstruction of a confocal image stack of the mouse submandibular gland. Acinar cell models are reasonably well developed (Vera-Sigüenza et al. 2018, 2019, 2020; Takano et al. 2021) and so we utilise these older models, with minor adjustments, to generate dynamic primary saliva flow to be used as input to the duct model. The acinar cell model output is the primary saliva flow rate and ionic concentrations, all given as functions of time. The time dependency occurs because the saliva flow increases upon nervous stimulation.

Here we extend these previous salivary gland duct models with an anatomically accurate salivary gland model with accurate individual cell geometry and duct network structure. We reconstruct the 3D geometry of the mouse submandibular gland based on a confocal image stack of the gland tissue. Our model is developed based on new experimental data on the expression levels of ENaC, TMEM16A, aquaporins and NaK ATPase in the ID and SD cells. Incorporating these new findings, our model demonstrates that the ID transports ion differently from SD. The model primary and final saliva compositions are fitted to the mouse salivary gland experimental results Mangos et al. (1973c) for both the unstimulated and stimulated conditions. We first fit the duct model to the unstimulated case as a benchmark and then to various stimulated flow rates to demonstrate model reliability.

## Experimental Methods and Data

### Methods

*Immunostaining of ion transporters* Submandibular glands (SMGs) from 3 to 6-month-old C57/BJ6 mice were fixed overnight in 4% paraformaldehyde. Tissue was embedded in paraffin and 5 µM sections were cut. Tissue was deparaffinised, rehydrated, and then, antigen retrieval was performed and optimised based on the antibody that was utilised. Sections were blocked in 10% normal donkey serum in PBS with 0.2% BSA or with M.O.M (Mouse on Mouse) Blocking reagent (MKB-2213-1; Vector Laboratories) for 1 h at room temperature. Following blocking, sections were incubated with primary antibodies overnight at 4 $^{\circ }$C [Aquaporin 5 (ab239904; Abcam), ENaC (PA1-920A; Invitrogen), NaK ATPase (ab2872; Abcam), NaK ATPase (ab7671; Abcam), TMEM16A (ab53212; Abcam)]. Secondary antibodies were applied the following day at a concentration of 1:500 and incubated for 1 h at room temperature [donkey anti-rabbit Alexa 488 (A-21206; Invitrogen) and donkey anti-mouse Alexa 594 (A-21203; Invitrogen)]. DAPI (62248; Thermo Scientific) was applied to the sections for 5 min at room temperature, and coverslips were mounted on to the slides using Immu-Mount (9990402; Epredia). Slides were imaged using an Olympus Scanning Confocal Microscope (FV1000) with a 60x Oil Objective (1.35 NA). Fiji was used to generate a 3D-projection of the image stacks and to add a scale bar.

*3D structure of the ducts* C57/BJ6 mice were anesthetised with ketamine (75–100 mg/kg)/xylazine (10 mg/kg) by IP injection and were placed on a specialised microscope stage on a water bath-warmed thermal pad. The submandibular gland excretory ductal openings are located slightly bilateral to the midline of the floor of the mouth, about 4–5 mm posterior from the lower incisors, underneath the tongue. To prevent salivation during the retrograde injection, an injection of sterile water containing 0.5 mg/kg of the muscarinic receptor antagonist, atropine, is delivered by IP injection to anesthetised mice 10 min prior to retrograde injection. The duct was injected with 20 µl of solution containing 50 µM sulphorrhodamine over 3–5 min. The mice were then imaged by multiphoton microscopy as previously described (Takano et al. 2021). 49 images of 1 µm spacing and 512 $\times$ 512 pixels resolution are obtained.

### Results

**Fig. 1:**
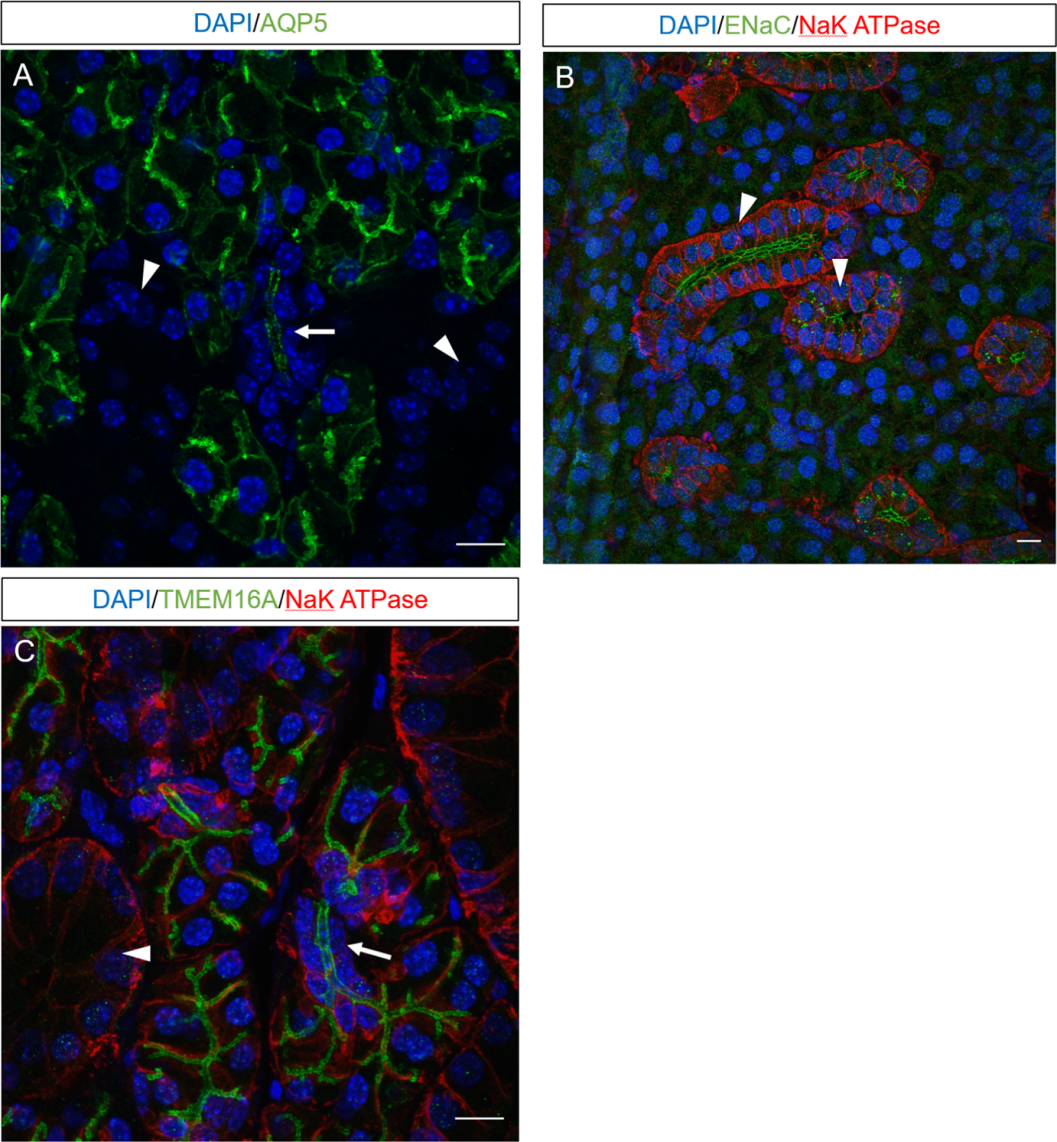
Immunofluorescent images of ducts within the submandibular gland. Sections of submandibular gland were labelled with antibody to AQP5 (**A**), ENaC (**B**), or TMEM16A (**C**) and counterstained with NaK ATPase (**B**, **C**). Nuclei were visualised using DAPI. Arrows identify IDs and arrowheads identify SDs within the representative sections (Color figure online)

Figure 1 shows the immuno-staining results from the mouse SMG ID and SD. It shows that aquaporin 5 is found in ID and not SD. ENaC channels are only expressed in the SD, with almost no ENaC in ID. TMEM16A are found in ID and not SD. NaK ATPase is found in ID and abundant in SD.

## Model Construction

### 3D Geometry

We constructed a realistic 3D mesh model of a branching segment of mouse SMG duct which included both ID and SD cells. The physical mesh model was used to determine the physical parameters and connectivity required by our numerical method.

#### Spatial Statistics

In general, realistic 3D reconstruction requires accurate physical dimensioning. We measured the sizes of salient features in an assortment of submandibular gland microscopy images for the purpose of compiling a table of spatial statistics (as shown in Appendix ). We consulted the measured values for guidance when reconstructing our full 3D duct structure.

#### Image Stacks and 3D Geometry

**Fig. 2:**
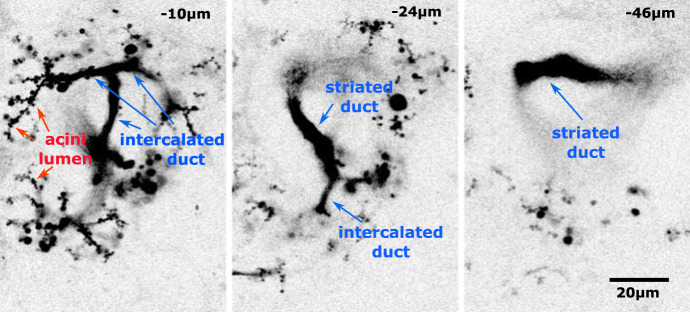
Three representative parotid gland microscopy image stack slices. Several acini lumen are indicated in red. ID and SD are indicated in blue. The total volume imaged was spanned by 49 images (Color figure online)

A microscopy image stack of an in vivo submandibular gland was the basis for our full 3D structural reconstruction. Figure 2 shows three of the forty-nine inverted intensity image slices that were used in the reconstruction. Note that the image stack traverses through 3D space in calibrated distance steps.

The experimental method used to acquire these images was designed to primarily highlight the duct tube-like inner structure. ID and SD segments were identified manually in each image. Note that the “up-stream” end of the duct connects to acinar lumens which were not required in the current reconstruction.

**Fig. 3:**
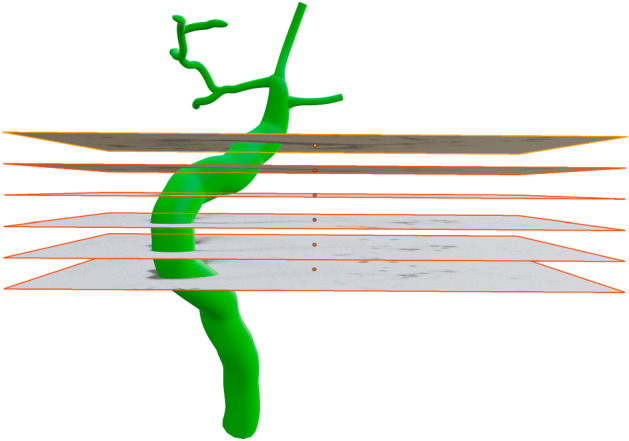
Six of the 49 images that were placed in 3D space using graphics software Blender. The resultant traced duct structure with appropriately sized radii is shown in green. All 49 images were used in the tracing process (Color figure online)

Using 3D graphics modelling software (Blender 2.93), we placed the image stack in calibrated space as shown in Fig. 3. Using the features identified in each image, we manually drew in Bezier curves that traced through the duct segments. For the final inner duct representation, tubes were generated graphically as a radial expansion around each of the traced Bezier curves using our tabulated spatial statistics for guidance with the radii. For example, as seen in Appendix , the SD average inner diameter is 8 µm, and the ID average diameter is 1.6 µm. The SD is roughly 80 µm long and the longest ID branch is roughly 45 µm long.

#### Growing Cells

Although our reference image stack was useful for inner duct reconstruction, it did not clearly show the intercalated and striated cell outlines that could have otherwise been used directly for cell reconstruction. Our solution to this problem was to “grow” realistic cells around our inner duct structure using physics simulation guided by our spatial statistics.

**Fig. 4:**
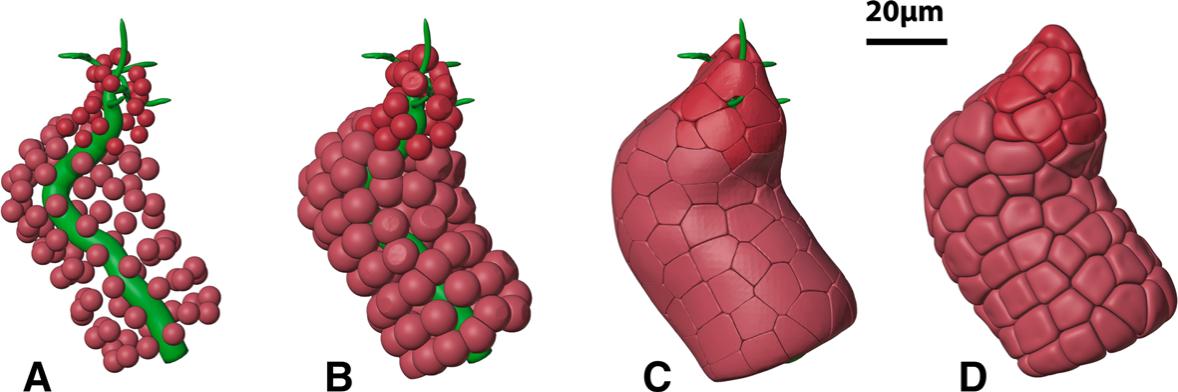
Growing duct cells around the inner duct constraint. **A** Cell seed placement. **B** Cell inflation with collision testing. **C** Tight cell packing constrained by the outer duct boundary. **D** The cells after final unconstrained spatial smoothing (Color figure online)

Firstly, we scattered a representative number of small ID and SD cell “seeds” around the inner duct as can be seen in Fig. 4A. Then, we used the standard physics simulation capabilities of Blender to perform inflation with collision testing to expand the cells. Note that the inflation is physically bounded by both the inner and outer duct diameter (Fig. 4B and C). Tight cell packing ensured that the (basolateral) surfaces of adjacent cells came into very close contact with each other, as is the case with real-world cells.

Additionally, since real-world cells do not have sharp edges, we employed a final spatial smoothing operation that was not constrained by the duct inner and outer boundaries, giving results as shown in Fig. 4D. These cells were saved as triangle mesh files available for our fluid-flow computations.

**Fig. 5:**
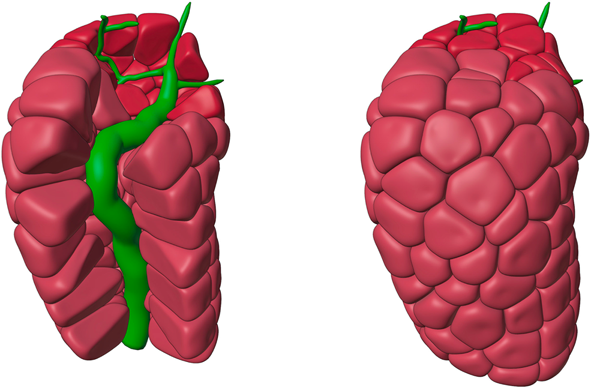
Duct cells. A 3D perspective image of the final cells is shown on the right-hand side. The cut-away view on the left-hand reveals tightly packed cells around the central inner duct

Figure 5 shows 3D perspective projection of the final cell collection. In the left-hand side of the figure, a number of the cells have been removed to reveal how the tightly packed cells wrap around the central inner duct tree.

Note that, in this reconstruction, the cells are the only real-world objects. The inner duct that we constructed is virtual, being simply the space that the duct cells enclose. However, there is valuable computational utility in the line structure of the duct, described as follows.

**Fig. 6:**
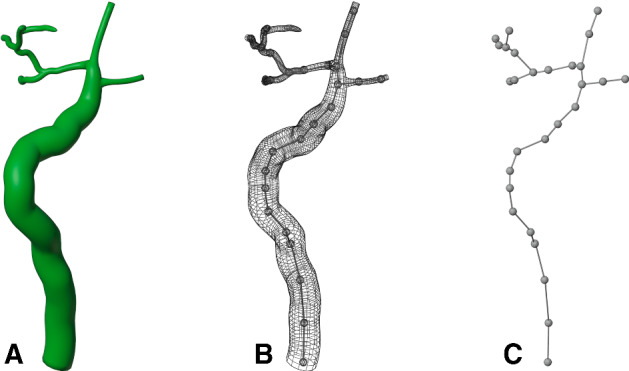
Duct discretisation. **A** The inner duct structural reconstruction. **B** Line segment placement through the duct centre. **C** The node-segment tree used for calculation purposes

#### Mesh Characterisation

The cell mesh files are stored in a standard triangle mesh graphics format which contains a list of triangle vertices (nodes) and triangle faces (surface elements) associated with the cell membrane (surface). The saliva modelling requires identification of the apical end of each cell. In the case of duct cells, the apical end of each cell is that portion of the cell surface that is in close contact with the (virtual) inner duct. To simplify this determination, we linearised the inner duct structure by partitioning it into a branching line-segment tree, as shown in Fig. 6.

The distance was determined from the centre of each cell mesh surface triangle to its nearest duct segment. If that distance was not much greater than the duct radius at that point, then the surface triangle was considered to belong to the cell apical region.

The virtual inner duct is partitioned for the computation of saliva concentrations. The numerical treatment of the inner duct and the duct–cell interface is described in Appendix .

### Model Construction

We construct a salivary minigland model that incorporates both saliva secretion and modification. It consists of several acini which extend into corresponding IDs which then join into one SD. Because the only interaction between the acini and the ducts is the primary saliva, the acinar cell model is solved separately from the duct model and the primary saliva output is used as the boundary condition for the duct model.

We modified the acinar cell model of Takano et al. (2021) to generate the primary saliva, while our duct model is a modified version of the minipig salivary gland duct model of Fong et al. (2017). The major changes from the model of Fong et al. (2017) are in the types and densities of ion transporters in both SD and ID cells. The changes are based on a literature review of mouse (instead of minipig) salivary gland information and new immunostaining evidence as shown in Sect. 2.

#### Acinar Cell Model

The previous 3D spatial temporal acinar cell model of Takano et al. (2021) describes how the calcium oscillations inside an acinar cell lead to primary saliva secretion. Upon neuronal stimulation, the acinar cell releases Ca$^{2+}$ from an internal store (the endoplasmic reticulum) near the apical membrane, which activates apical Cl$^-$ channels thus allowing a flux of Cl$^-$ into the lumen. Following the anion movement, Na$^+$ enters the lumen through the tight junctional pathway. These ionic currents raise the osmolarity of the luminal fluid, and thus draw water out of the cell through aquaporins on the apical membrane and tight junctions between cells (Nauntofte 1992). The fluid mixture secreted is the primary saliva.

However, the model of Takano et al. (2021) produces primary saliva that is not exactly compatible with the input requirement of the duct model; it contains only 3 ion species, Na$^+$, K$^+$ and Cl$^-$, whereas the duct model also requires HCO$_3^-$ and H$^+$. In order for the two to be compatible, we modified the acinar cell model to produce HCO$_3^-$ and H$^+$ in the primary saliva. HCO$_3^-$ is produced through the action of carbonic anhydrase and secreted through TMEM16A. TMEM16A is known to be permeable to both Cl$^-$ and HCO$_3^-$, with a P$_\mathrm{HCO3}$/P$_\mathrm{Cl}$ ratio of 0.3 when there is no intracellular Ca$^{2+}$ (Jung et al. 2013). H$^+$ is introduced through a bicarbonate pH buffering reaction by carbonic anhydrase in the luminal compartment.

Upon addition of HCO$_3^-$ and H$^+$, the total osmolarity of the ions in the primary saliva increases. To maintain the overall osmolarity of the primary saliva, we reduce the digestive protein concentration from 50 to 10 mM. We also adjust certain model parameters so that the Na$^+$, K$^+$ and Cl$^-$ concentrations fit more closely to the measurements in Mangos et al. (1973c). Table 1 shows the ionic composition of the unstimulated primary saliva from the model of Takano et al. (2021), the updated model in this paper and the measurement from Mangos et al. (1973c). Table 2 lists the modified parameter values in the updated model.

**Table 1:** Table of the comparison between the unstimulated primary saliva produced by the model of Takano et al. (2021) and the updated model in this paper

Variable	Unit	Model of	Updated	Measurement
		Takano et al. (2021)	model	Mangos et al. (1973c)
Na$^+$	mM	117.25	136.9	151.6
K$^+$	mM	6.41	6.9	5.1
Cl$^-$	mM	123.65	115.4	124.6
HCO$_3^-$	mM	–	28.4	34.1$^\mathrm{a}$
pH		–	7.11	–
Digestive protein	mM	50	10.4	–
Osmolarity	mM	298	298	297.4
Primary saliva flow rate	µm$^3$/s	14.43	11.91	–$^\mathrm{b}$

The model modification is done so that the primary saliva composition is more similar to that measured in Mangos et al. (1973c)

$^\mathrm{a}$The bicarbonate concentration value is not measured in Mangos et al. (1973c), but derived from Na$^+$, K$^+$, Cl$^-$ concentrations to achieve electroneutrality.

$^\mathrm{b}$Measured flow rate can be found in Mangos et al. (1973c). The number is not included here because it is in the unit of µL per minute per gram of gland weight.

The acinus model of Vera-Sigüenza et al. (2020) has shown that the spatial distribution and the shape variation of acinar cells in a cluster do not affect the saliva generation rate. Therefore, it is acceptable to replace the saliva generated by a cluster of 7 acinar cells with 7 times that of one acinar cell. The primary saliva entering the ducts is generated on the mesh of cell 4 of the 7 cell acinus model in Vera-Sigüenza et al. (2020).

**Table 2:** The modified parameters of the model of Takano et al. (2021) and the updated acinar cell model

Parameter	Model of Takano et al. (2021)	Updated model	unit
Digestive protein	50	10	mM
Apical $\mathrm G_K$	0.1672	0.0836	nS
Luminal bicarbonate buffering $\mathrm k_p$	0.132	0.1056	s$^{-1}$
P$_\mathrm{HCO3}$/P$_\mathrm{Cl}$	0	0.3	–

#### SD Cell Model

The SD cells absorb Na$^+$ through ENaC channels (Catalán et al. 2010). With the high luminal and low cellular Na$^+$ concentrations and the negative apical membrane potential, the duct cells are effective at Na$^+$ absorption and, at the same time, require fast Na$^+$ removal at the basolateral side. Immunostaining shows strong expression of the NaK ATPase on the basolateral membrane of the SD cell (Winston et al. 1988), which is consistent with this requirement.

**Fig. 7:**
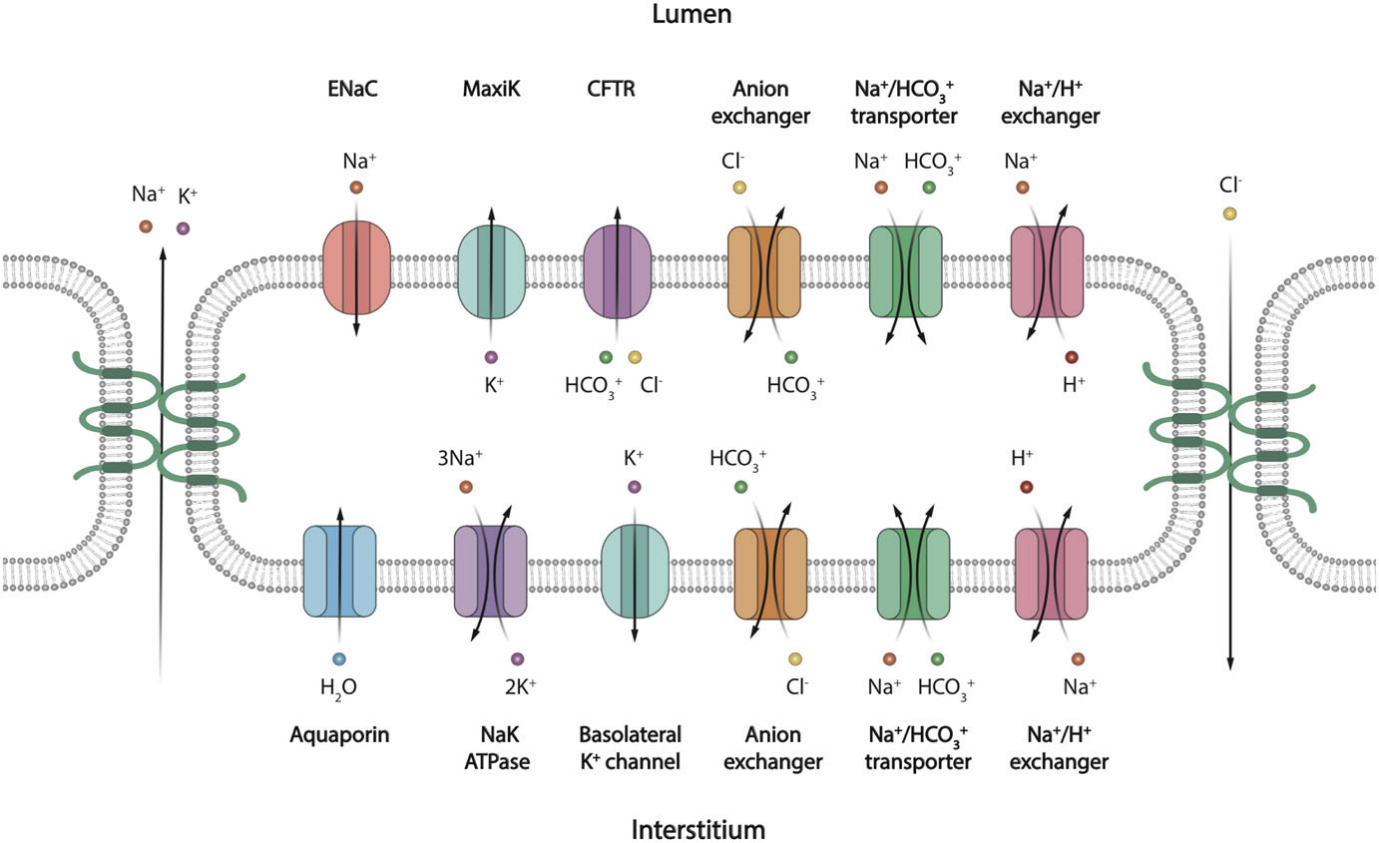
Schematic diagram of a salivary gland SD cell, showing the types of ion and molecule transporters on the apical and basolateral membrane. The apical membrane faces the lumen and the basolateral membrane faces the interstitium. The tight junctions between cells allow the passage of Na$^+$, K$^+$ and Cl$^-$. The directions of all arrows are indicative of the actual movement of ions in the majorities of the SD cells along the duct, based on the simulation results in the result section (Color figure online)

The other ion transporters we include in our SD cell model are largely similar to those in the model of Fong et al. (2017). Figure 7 shows the transport proteins in the SD cells. The apical membrane of the SD cells contains ENaC channels (Schneyer 1970; Catalán et al. 2010), CFTR channels (Catalán et al. 2010), Maxi-K channels (Catalán et al. 2014; Nakamoto et al. 2008), Na$^+$/H$^+$ exchangers (Park et al. 1999; Zhao et al. 1995; He et al. 1996), Na$^+$/HCO$_3^-$ cotransporters (Luo et al. 2001; Li et al. 2006) and anion exchangers (Zhao et al. 1995; Shcheynikov et al. 2008). On the basolateral membrane, there are NaK ATPase (Smith et al. 1987; Winston et al. 1988), ATP-sensitive K$^+$ channels (Zhou et al. 2010; Liu et al. 1999), Na$^+$/H$^+$ exchangers (Zhao et al. 1995), anion exchangers (Zhao et al. 1995), Na$^+$/HCO$_3^-$ cotransporters (Li et al. 2006; Luo et al. 2001) and aquaporins (Delporte et al. 2016; Matsuzaki et al. 2012).

The detailed mathematical formulations of the transporter rates are described in Appendix . All of the transporter density coefficients are fitted to match mouse measurements (Mangos et al. 1973c), with the parameter values shown in Appendix .

#### ID Cell Model

**Fig. 8:**
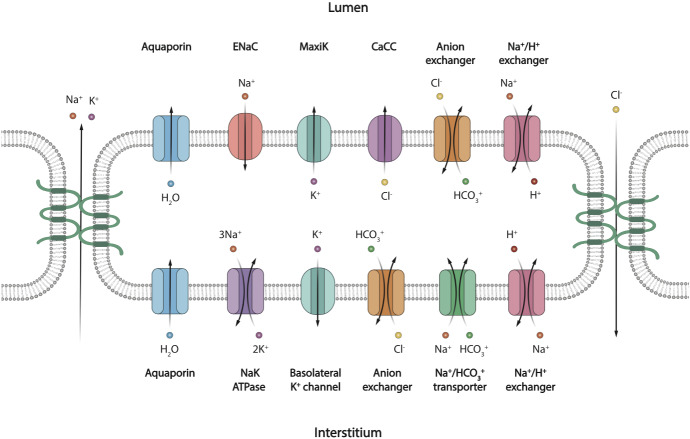
Schematic diagram of a salivary gland ID cell (Color figure online)

There is little information in the literature about which ion transporters are expressed in ID cells. Figure 1 shows that AQP5 are expressed in the apical membranes (Matsuzaki et al. 2012; Delporte et al. 2016), as are TMEM16A channels, whilst there are very few ENaC expressed. Figure 8 shows the transporters in our ID cell model.

The traditional view of the ID is that it absorbs Na$^+$ and Cl$^-$ like the SD and is impermeable to water at the apical membrane (Varga 2012). However, our data contradict this view. The low level of apical ENaC staining indicates that the cell has low Na$^+$ absorbing capacity. Given that the ENaC channel is the main pathway for NaCl absorption in the SD (Catalán et al. 2010), we conclude that the ID does not absorb much Na$^+$. The existence of apical CaCC suggests that the ID secretes Cl$^-$ upon nervous stimulation, which suggests also that the high osmolarity of the primary saliva is preserved across the ID. Aquaporins have been found on the apical membrane of the ID (Matsuzaki et al. 2012), which means it has substantial water permeability. The water permeability, together with the higher luminal than cellular osmolarity, suggests that the ID is water-secreting and is sensitive to nervous stimulation. The ID is a short section of duct between the acinus and the SD. There is evidence that ID cells differentiate to either acinar cells or granular duct cells in rat submandibular gland (Man et al. 2001), and thus, it is plausible that the ID forms a transition region between the acinus and the SD, and that ID cells have properties of both cell types.

### Duct Model Formulation

We assume each duct cell is a well-mixed compartment and represent each ion concentration with one average value. We model 5 ion species: Na$^+$, K$^+$, Cl$^-$, HCO$_3^-$ and H$^+$. We use [N]$_\dagger$ to represent the concentration of ion N in compartment $\dagger$, where $\dagger$ = C for the cellular compartment, $\dagger$ = L for the luminal compartment, and $\dagger$ = I for the interstitial compartment. For example, $[\mathrm{Cl^-}]_C$ is the cellular Cl$^-$ concentration.

In a cell, the rate of change of the number of moles of an ion is given by

1 $$\begin{aligned} \frac{d([\mathrm{N}]_C w_C)}{dt} = A_A J_a + A_B J_b + J_\mathrm{buf}, \end{aligned}$$

where $w_C$ is the cell volume, *t* is time, $A_A$ and $A_B$ are the cell apical and basolateral membrane areas, in units of µm$^2$, $J_a$ and $J_b$ are the average per unit area fluxes of ion N across the apical and basolateral membrane, respectively, in units of mol/(s µm$^2$), and  is the ion fluxes from buffering reaction in the cell, in units of mol/s.

The luminal concentrations depend on time and the distance (*x*) along the duct. The saliva flow is modelled as 1D fluid flow with variable cross-sectional area. Physically, ions move along the duct via convection and diffusion. Computation demonstrates that for salivary gland ducts, diffusion does not significantly affect the steady-state distribution of ion concentrations (computation not shown here), and thus, we assume diffusion is negligible. We also assume the hydrostatic pressure field within the duct is negligible because there is no known quantitative measurement of the contraction force of the myoepithelial cells that assists fluid movement. Since the primary purpose of our model is to determine how ions are transported downstream along the duct, we model the saliva flow profile using the 1D convection PDE

2 $$\begin{aligned} \frac{\partial ([\mathrm{N}]_L A)}{ \partial t} + \frac{\partial ([\mathrm{N}]_L V)}{\partial x} = -J A_L, \end{aligned}$$

where *A* is the cross-sectional area of the duct, which is a function of x, *V* is the volumetric flow rate of saliva, in units of µm$^3$/s, *J* is the apical ion flux per unit area, in units of mol/(s µm$^{2}$), and $A_L$ is the lumen surface area per unit length, in unit of µm.

The spatial dimension *x* is the distance along the ductal lumen centreline. We numerically solve the PDE by discretising the lumen into 1 µm long segments. Assuming each segment is a well-mixed compartment, we assign 5 concentration variables to each lumen segment. The luminal discretisation splits the apical membrane of each cell into several 1-µm wide strips. The ion flux through each membrane strip is computed based on the strip surface area and the cellular and luminal concentrations on either side of the strip. For a detailed description of the numerical treatment of the duct discretisation, see Appendix . The total ion flux into a lumen segment is the sum of the fluxes from all adjacent apical membrane strips.

Using the spatial discretisation, we approximate the *x* derivative with an upwind finite differences scheme, which produces a system of ODEs, one for each lumen segment. The ODEs are

3 $$\begin{aligned} \frac{d [\mathrm{N}]^i_L A_i}{d t} + \frac{[\mathrm{N}]^{i}_L V^i - [\mathrm{N}]^{i-1}_L V^{i-1}}{\Delta x} = -J^i A_L^i, \end{aligned}$$

where [N]$^i_L$ represents the concentration of ion N in the *i*th lumen segment, $V^{i-1}$ and $V^{i}$ are the volumetric flow rates into and out of the lumen segment, respectively, $\Delta x$ is the discretisation step size (1 µm), $J^i$ is the total ion flux across all apical membrane strips surrounding the segment, and $A_L^i$ is the surface area of the *i*th segment.

From Eqs. 1 and 3, we obtain a system of ODEs for each cell and for each lumen segment. The detailed equations for ion transporter fluxes, cell volume and water flow can be found in Appendix . The composition of one cell is dependent on its upstream cells via the luminal composition. To solve the equations we use MATLAB ODE solver ode15s, with an absolute tolerance of $10^{-10}$ and a relative tolerance of $10^{-9}$.

## Model Results

The 3D duct reconstruction is based on the tissue images of the mouse submandibular gland. The immunostaining results of ion transporters in the ducts are based on experiments on a range of animals and gland types, as described in Sect. 3.2.2. When we fit the model results to saliva measurement, we find it is simpler to fit to the mouse parotid gland experiments. In Discussion, we discuss the difficulties of fitting to the submandibular gland data and propose a way to achieve the fit.

### Unstimulated Case

The unstimulated primary saliva generated by the updated acinar cell model is shown in Table 1. We run the duct model to equilibrium with the unstimulated primary saliva as a constant input.

**Fig. 9:**
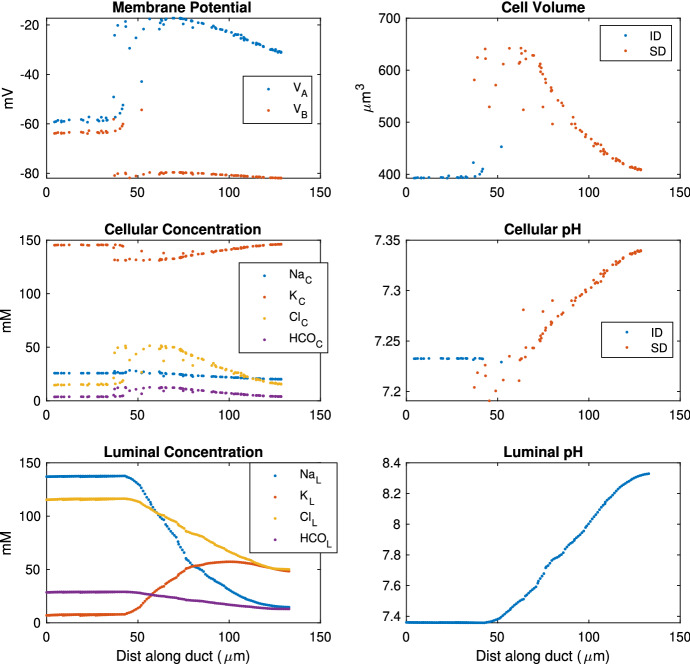
The steady-state solution of the duct model with unstimulated primary saliva flow. The *x* axis is the distance along the duct, starting from the acinus farthest from the duct outlet. On the x axis, the ID is on the left (0 to 45) and SD on the right (45 to 130). For the cellular concentrations, each data point represents a discrete cell, for the lumen, each data point is one lumen discretisation segment, most of which are 1 µm long. The ID consists of several branches which join together and become the SD, and thus, the ID data points overlap (Color figure online)

The steady-state solutions of the cellular and luminal concentrations along the duct are plotted in Fig. 9. In the duct cells, [Na$^+]_C$ and [K$^+]_C$ are consistently maintained at physiological values, as is the intracellular pH. [Cl$^-]_C$ and [HCO$_3^-]_C$, on the other hand, vary to some extent. The ID has a small transepithelial potential of 5 mV, while the SD has a much larger one. Since the primary saliva has a similar ion composition as the interstitium, it is anticipated that the transepithelial potential at the proximal end should be small. The transepithelial potential at the distal end of the duct is high (60 mV), which is consistent with experimental measurements of the main excretory duct of rat submandibular gland (50 and 82 mV) (Schneyer 1970). The ID cell volumes ($\sim$ 400 µm$^3$) are smaller than the SD (470$\sim$890 µm$^3$ ), which is consistent with that estimated in the tissue images (Appendix ). Table 3 compares the modelled cellular ion concentrations to the experimental measurements. Overall the duct model is in good agreement with the physiological properties of the duct cells.

**Table 3:** The model results for the duct cells compared against experimental measurements

Variable	Model	Measurement	Unit	Source
$[\mathrm{Na}^+]_C$	20–25	17	mM	Zhao et al. (1995)
$[\mathrm{K}^+]_C$	131–146	140	mM	Lodish (2016)
$[\mathrm{Cl}^-]_C$	14–51	22	mM	Lee et al. (1999)
$[\mathrm{HCO}_3^-]_C$	3–12		mM	
$\mathrm{pH}_C$	7.2–7.34	7.2–7.4	mM	Zhao et al. (1995)
ID $w_C$	400	$\sim$ 200	µm$^3$	Appendix
SD $w_C$	400–890	$\sim$ 1000	µm$^3$	Appendix
Distal ($V_A - V_B$)	50	50–82	mV	Schneyer (1970)

**Table 4:** Comparison between the unstimulated final saliva measurement and model results

Variables	Model	Measurement	Unit	Source
[Na$^+]_L$	12	13	mM	Mangos et al. (1973c)
[K$^+]_L$	57	62	mM	Mangos et al. (1973c)
[Cl$^-]_L$	54	54	mM	Mangos et al. (1973c)
[HCO$_3^-]_L$	14	10	mM	Mangos et al. (1973c)
pH	8.3	8.18		Catalán et al. (2010)

In the lumen, [Na$^+]_L$ and [Cl$^-]_L$ drop and [K$^+]_L$ rises as required. The saliva at the end of the duct is considered to be the final saliva. Table 4 shows the modelled final saliva compared to the mouse parotid gland measurements. The model achieves a good agreement with the experiments for all ion concentrations.

The ID cell apical membrane is permeable to water, and thus, water is secreted. The primary saliva flow from all four acini is 417 µm$^3$/s. The total final saliva flow out of the SD is 419 µm$^3$/s, which results in an ID water secretion rate of 2 µm$^3$/s.

**Fig. 10:**
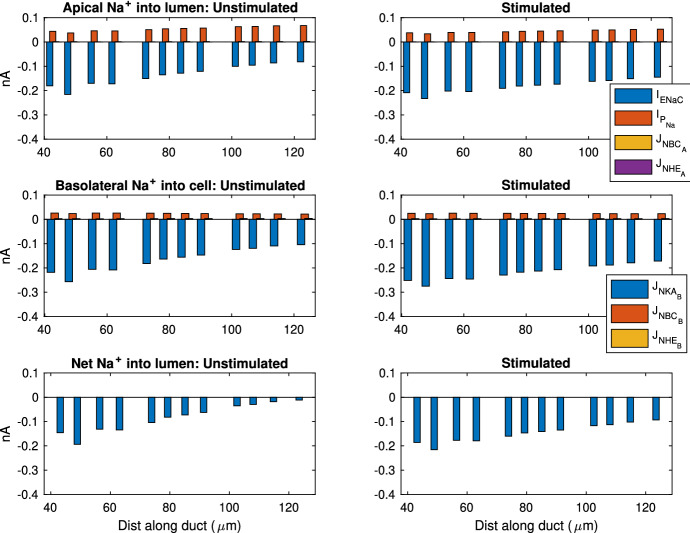
This plot shows a selection of SD cells and the Na$^+$ flux contributed by each ion transport mechanism. The SD cells shown here are randomly selected so the entire duct is sampled evenly. The left panels show the fluxes in unstimulated gland, while the right panels show the stimulated. The stimulated data are collected at 400 s after stimulation is turned on. Top panels show Na$^+$ flux into the lumen from cell, and a negative value indicates flux into cell. The middle panels show flux across the basolateral membrane, and a negative value means flux into the interstitium. The bottom panels show the overall Na$^+$ flux into the lumen, which is the net flux of all apical fluxes in the top plot.  is the current across ENaC,  is the paracellular current,  is the flux through the apical Na$^+$/HCO$_3^-$ cotransporter,  is the flux through the apical Na$^+$/H$^+$ exchanger, and  is the flux through the basolateral NaK ATPase (Color figure online)

**Fig. 11:**
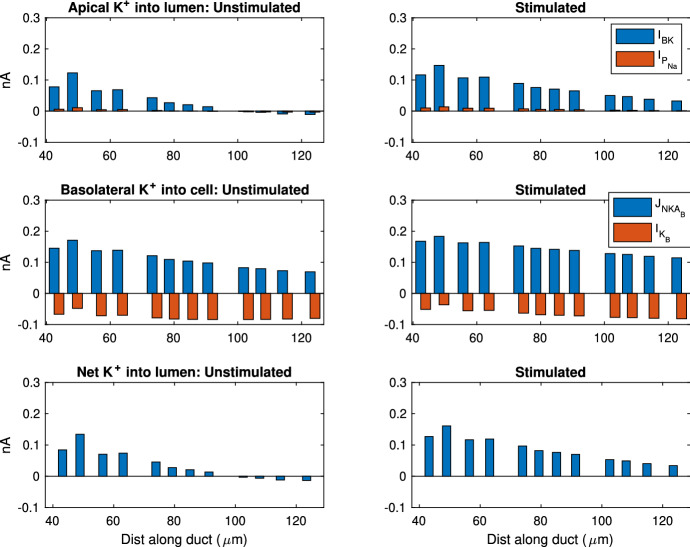
This plot shows a selection of SD cells and the K$^+$ transport rates contributed by each ion transport mechanism. The SD cells shown here are randomly selected so the entire duct is sampled evenly. The left panels show the fluxes in unstimulated gland, while the right panels show the stimulated. The stimulated data are collected at 400 s after stimulation is turned on. Top plots show K$^+$ flux into the lumen from cell, and a negative value indicates flux into cell. The middle plots show flux across the basolateral membrane, and a negative value means flux into the interstitium. The bottom plots show the overall K$^+$ flux into the lumen, which is the net flux of all apical fluxes in the top plot.  is the current across apical maxi-K$^+$ channels,  is the paracellular current,  is the flux through the basolateral NaK ATPase and  is current across the basolateral K$^+$ channels (Color figure online)

**Fig. 12:**
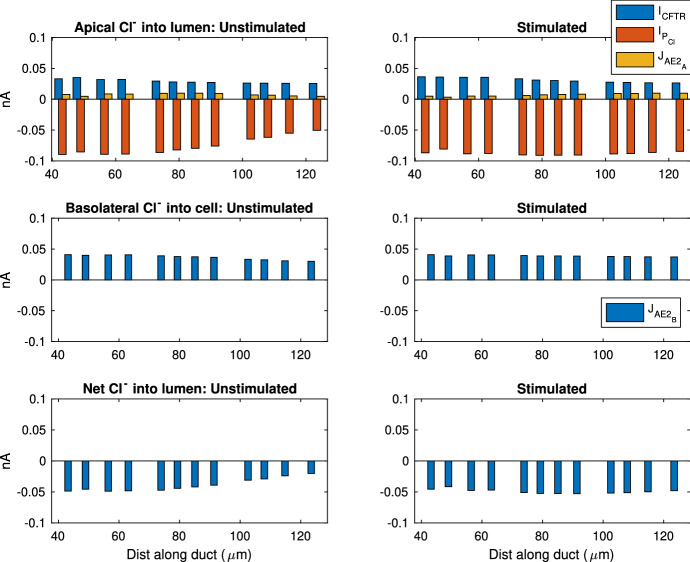
This plot shows a selection of SD cells and the Cl$^-$ transport rates contributed by each ion transport mechanism. The SD cells shown here are randomly selected so the entire duct is sampled evenly. The left panels show the fluxes in unstimulated gland, while the right panels show the stimulated. The stimulated data are collected at 400 s after stimulation is turned on. Top plots show Cl$^-$ fluxes into the lumen from cell, and a negative value indicates fluxes into cell. The middle plots show fluxes across the basolateral membrane, and a negative value means flux into the interstitium. The bottom plots show the overall Cl$^-$ flux into the lumen, which is the net flux of all apical fluxes in the top plot.  is the current across apical CFTR channels,  is the paracellular current,  and  are the fluxes through the apical and basolateral anion exchangers, respectively (Color figure online)

The model reveals how ions are transported across the SD epithelium. Figure 10 shows the direction and magnitude of Na$^+$ transport at different positions along the SD by sampling some SD cells. In general, the Na$^+$ is pumped out of the cell by the basolateral NaK ATPases. The low [Na$^+]_C$ results in an electrochemical gradient across the apical membrane which draws Na$^+$ into the cell via the ENaC channel. At the beginning of the SD, the ENaC flux is high due to the high [Na$^+]_L$ and thus high electrochemical gradient. The ENaC flux reduces as the [Na$^+]_L$ reduces. Figure 11 shows the same information for K$^+$ transport. The K$^+$ enters the cell through the basolateral NaK ATPases, and leaves through the apical and basolateral K$^+$ channels (I and I, respectively).

In the SD, Cl$^-$ is extracted from the saliva. Figure 12 shows that Cl$^-$ enters the cell through the basolateral AE, and leaves through both the CFTR and apical AE. The overall Cl$^-$ movement from the lumen to the interstitium occurs through the paracellular pathway. To justify this, we refer to Fig. 7. Since the only two electrogenic ion transporters on the basolateral membranes both pump positive charges outwards, positive charges should enter the cell on the apical membrane to maintain electroneutrality. There are 3 major electrogenic transporters on the apical membrane, the ENaC, the K$^+$ channel and the CFTR. The ENaC channel and apical K$^+$ channel transport positive ions opposite ways with roughly equal flux magnitudes, as observed by the similar amount of change in salivary [Na$^+]_L$ and [K$^+]_L$. Therefore, the CFTR channel must transport Cl$^-$ outwards to balance the intracellular charge. There is a net positive charge flux from lumen to interstitium through the transcellular pathway. In order to maintain electroneutrality of the interstitium, there is an equal and negative flux through the paracellular pathway. The paracellular Cl$^-$ flux from lumen to interstitium balances the charge and also results in a net reduction of [Cl$^-]_L$.

### Stimulated Case

#### Acinar Cell Model Results

**Fig. 13:**
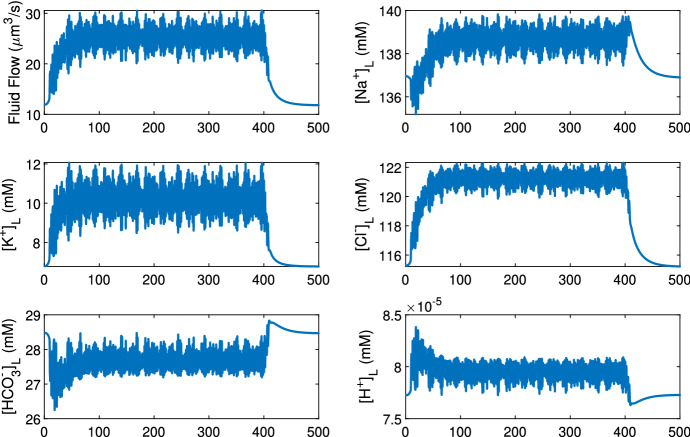
The time series data of the primary saliva output from the acinar cell model, at a low stimulation level. The stimulation is turned on at time 0 s and off at 400 s. The saliva flow rate and ion concentrations oscillate due to the calcium oscillations induced by the stimulation. The mean values of all variables rise first and then flatten at around 50 s. All values return to unstimulated levels 100 s after stimulation is turned off

**Fig. 14:**
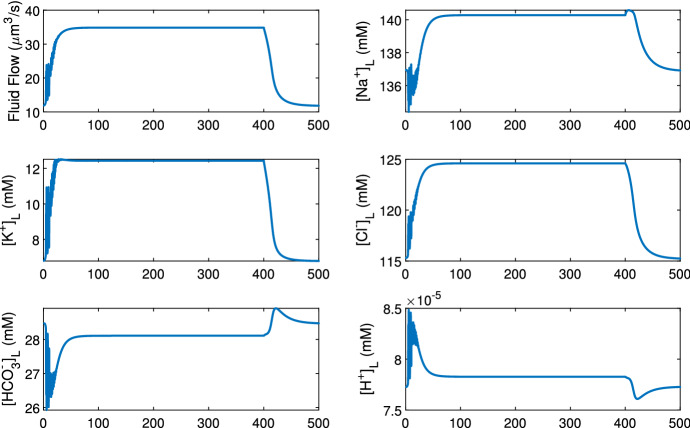
The time series data of the primary saliva output from the acinar cell model, at a high stimulation level. The stimulation is turned on at time 0 s and off at 400 s. The saliva flow rate and ion concentrations oscillate at the onset of the stimulation. However, as the cellular calcium concentration increases to a plateau due to high stimulation, the oscillation disappears and the flow rate and corresponding concentrations remain flat

The mouse salivary gland can secrete saliva up to 100 times faster under pilocarpine stimulation (Mangos et al. 1973c). In the acinar cell model of Takano et al. (2021), water secretion is driven by calcium oscillations inside the cell. Upon stimulation, [Ca$^{2+}]_C$ increases in an oscillatory manner. Higher [Ca$^{2+}]_C$ activates the Cl$^-$ channels which causes ion and water secretion, and thus, both water flow and ion concentrations fluctuate. Figure 13 shows the acinar cell model outputs under a low stimulation, where the fluctuation is present throughout the duration of the stimulation. Figure 14 shows a case with a higher stimulation where the flow rate and ion concentrations reach a plateau.

The flow fluctuation occurs out of phase from cell to cell, and thus, the combined flow profile of a whole acinus fluctuates at smaller amplitude than that of a single acinar cell (computational results not shown here). However, the fluctuation amplitude does not affect the duct model output. This is shown by smoothing out the fluctuation of the primary saliva which still generates the same duct model outcome (computation results also not shown). Therefore, the fluctuating flow profile can be replaced with a smooth profile representing the average flow.

**Fig. 15:**
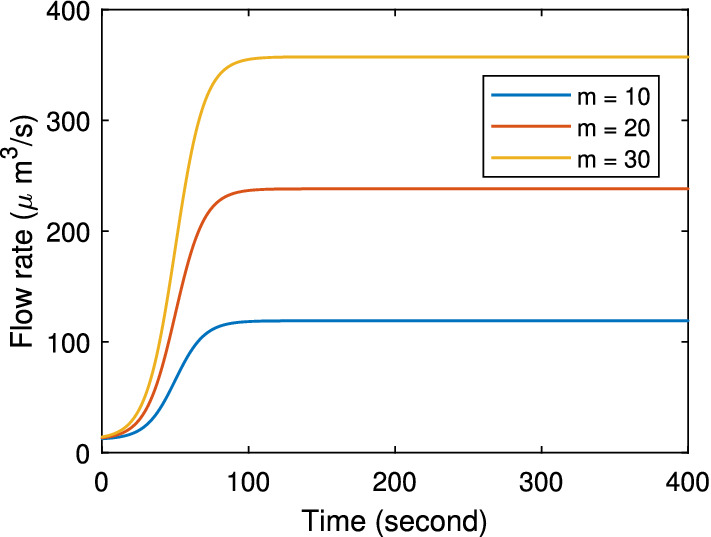
A sigmoid function is used to approximate the increase of an acinar cell saliva flow under different stimulation levels. *m* is the multiplier of the unstimulated flow rate, which determines the stimulated flow rate. We assume saliva flow drops back to initial unstimulated level after 400 s (Color figure online)

At higher stimulation, the acinar cell model predicts a higher flow rate and primary saliva osmolarity. The acinar cell model can generate up to 4 times the unstimulated flow rate. The relationship between osmolarity and flow rate is linear. At 4 times the basal flow, the osmolarity increases from 297 to 330 mM. According to Mangos et al. (1973c), when stimulated by pilocarpine, the mouse salivary gland can secrete up to 100 times faster than the unstimulated case. What is more, at the maximum flow rate of the acinar cell model, the osmolarity remains almost unchanged (Mangos et al. 1973c). Therefore, when modelling a high flow rate, we assume a constant primary saliva composition. We construct a sigmoid function to approximate the increasing saliva flow,

4 $$\begin{aligned} Q(t) = \dfrac{Q_\mathrm{basal}(m - 1)}{1+e^{0.1(t-50)}}+Q_\mathrm{basal}, \end{aligned}$$

where *Q*(*t*) is the stimulated secretion rate with respect to time, $Q_\mathrm{basal}$ is the unstimulated secretion rate and *m* is the flow rate multiplier. Figure 15 shows the flow profiles for different multipliers. In the experiments, the saliva at different stimulation levels is collected for 3–10 min (Mangos et al. 1966). Therefore, we consistently use a stimulation duration of 400 s throughout this paper.

**Fig. 16:**
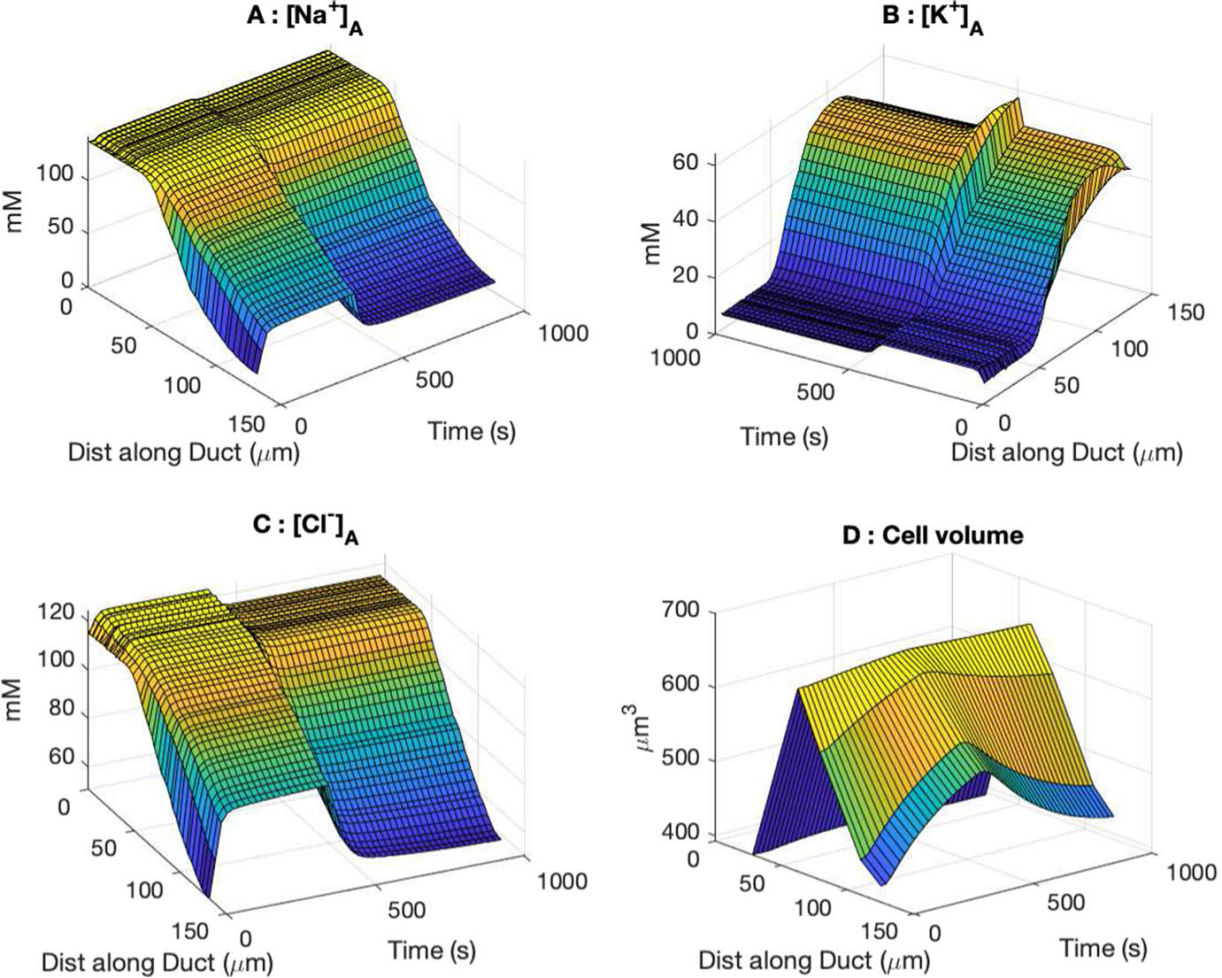
The temporal response of the duct to the stimulated primary saliva input as in Fig. 14. Plots A, B and C show how the saliva ion concentrations along the duct change over time. The saliva stimulation is switched on at *t* = 0 and switched off at *t* = 400 s. The plots show that [Na$^+$], [K$^+$] and [Cl$^-$] do not change significantly in the ID (situated less than 50 µm along the duct) but vary much more in the SD. This is because the SD is the main site of ion transport, and an increased flow makes it hard to remove ions fast enough thus the concentrations rise. They return to the unstimulated states very soon after the stimulation is off and the flow rate subsides. Plot D shows the cell volume change of 4 cells at different positions along the duct. It shows the volume of the two upstream cells remains the same across time, whereas the two downstream cells swell with stimulation. The effect reverses when the stimulation is removed (Color figure online)

#### Duct Model Results

In our model, we assume that duct cells do not directly respond to the neuronal stimulation, but indirectly respond via the stimulated primary saliva flow. Starting from the unstimulated equilibrium solution, we model the duct response by feeding into the duct the stimulated primary saliva flow, as shown in Fig. 14. Figure 16 shows the transient response of the duct to the stimulated saliva flow. The stimulation is on during the first 400 s. Since the ID does not perform much ion exchange, the saliva composition remains largely the same during stimulation. In the SD, a high saliva flow supplies NaCl faster than the duct cells can remove it, and thus the [Na$^+]_L$ and [Cl$^-]_L$ rise, whereas the [K$^-]_L$ drops. The effects quickly reverse when the stimulation is turned off at 400 s.

The stimulation also affects the duct cell volume. Plot D of Fig. 16 shows the cell volume change of 4 cells along the duct. The volume of the two cells close to the acinus remains almost the same, whereas the two cells near the end of the SD swell slowly during stimulation and the effect reverses when the stimulation is turned off. The cell volume reacts to the luminal fluid the cell is exposed to. In the ID, saliva composition remains the same and so does the cell volume. In the SD, the change in luminal fluid ion composition causes the cell volume to change. It appears that the cell swells when it needs to perform ion transport faster, since the high influx of Na$^+$ raises the cellular osmolarity, which in turn drives water into the cells through basolateral membrane. This is also consistent with the steady-state solution where the SD cells closer to the ID have a larger volume than those near the end.

The above result uses one saliva flow profile. Since final saliva composition is a function of the flow rate, we aim to fit to a range of flow rates, where the flow curves are artificially generated sigmoid functions. In the model, we record the final saliva at 400 s after stimulation turns on. Note that the model system has not reached temporal equilibrium at that point. The results we collect are at a transient state of the model.

**Fig. 17:**
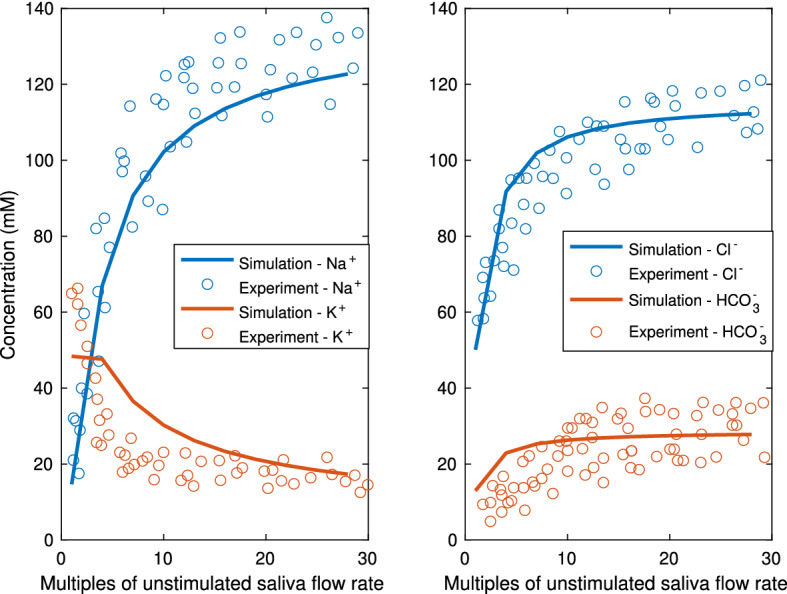
This plot is an overlay of the simulation results on an experimental dataset from Mangos et al. (1973c). It shows the mouse parotid gland final saliva composition as a function of the saliva flow rates. In the experiments, the saliva is collected for 3–10 min upon pilocarpine stimulation. In the model results, the final saliva composition is recorded when stimulation is on for 400 s. The model results (in lines) falls within the range of the experimental measurements (circles) throughout the whole range of flow rates (Color figure online)

Mouse salivary glands measurements (Mangos et al. 1973c) show that the final saliva composition depends on the flow rate. A faster flow rate raises the [Na$^+]_L$ and [Cl$^-]_L$ and lowers the [K$^+]_L$ in the final saliva. Figure 17 shows the measured final saliva at variable flow rates, with the model simulation results overlaid on top. The model results fall well within the experimental range, thus showing the model can reproduce the experiments for the whole flow rate range.

### Model Simplification

It has been shown in the acinar cell model of Vera-Sigüenza et al. (2020) that the total saliva secretion of the cells in an acinus can be well approximated by the saliva secretion of one cell multiplied by the number of cells in the acinus. It appears that in acinus modelling, the cell spatial distribution is not an important factor that affects saliva secretion. By omitting the geometric information of the cells, we can greatly simplify the modelling process. Therefore, we apply the same idea to the duct cell model. Instead of modelling each individual duct cell, we group all the ID cells together, forming a single ID cell compartment. The same goes for the SD cells. The duct lumen is divided into two compartments, one for the ID and one SD. The SD lumen compartment has the total SD lumen volume and length and similarly for the ID compartment. The same ion transporter parameters are used for the simplified compartments.

**Fig. 18:**
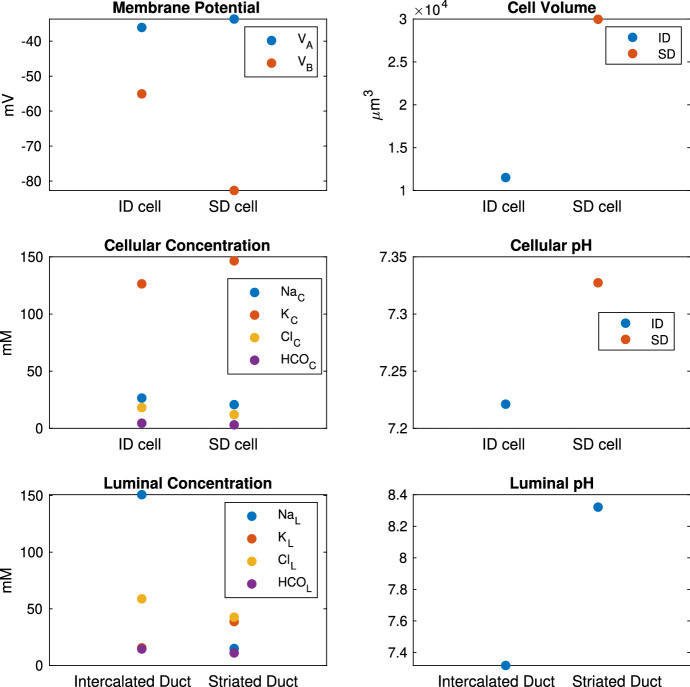
This plot shows the temporal steady-state result of the simplified duct model. All SD cells are grouped together into one SD cell compartment and so are the ID cells. The top 4 panels show the properties of the ID and SD cell compartment. Each compartment has the aggregated volume of all the ID or SD cells. The duct is divided into 2 sections (ID and SD), and the bottom 2 panels show the saliva composition of the two sections. The results are to be compared with the full model results in Fig. 9. Comparison shows that the simplified model gives accurate results for all cellular and luminal variables

The result of the simplified model is plotted in Fig. 18. Instead of a profile along the lumen, the model only produces two data points, one for ID and one for SD. We compare the simplified model with the full model results in Fig. 9 for its accuracy. The membrane potentials and the intracellular and luminal compositions of the simplified model mostly reflect the average ductal and cellular values in the full duct. The cell volume in the simplified model roughly equals the total volume of all ID or SD cells. The luminal composition in SD is considered the final saliva composition, which reasonably matches the experimental measurements. Therefore, the simplified model produces results with sufficient accuracy while greatly reducing the computational complexity. This indicates that the final saliva composition is insensitive to the details of the gland structure.

**Fig. 19:**
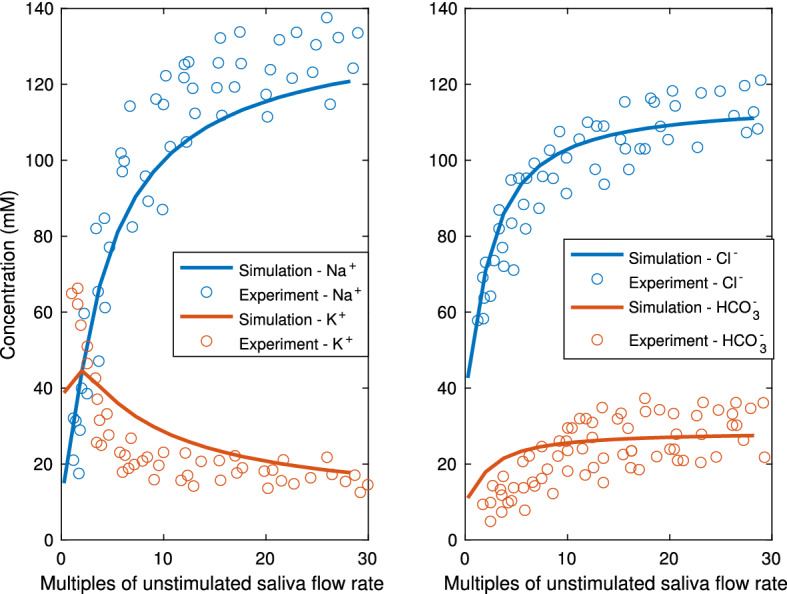
This plot is the final saliva composition produced by the simplified model over a range of flow rates. The model results lie well within the range of experimental data

We then run the simplified model with various flow rates as described in the full model. We treat the saliva in the SD lumen compartment as the final saliva and plot the concentrations against the flow rates, in Fig. 19. The simplified model captures the relationship between final saliva composition and flow rate very well.

## Discussion

In this paper, we develop a salivary minigland model that integrates the acinus and the duct. The two parts are solved separately and the acinus model output is the duct model input. We modify the acinus model of Takano et al. (2021) to generate the input primary saliva, whereas the duct model is an extensive re-development of the model of Fong et al. (2017). The anatomically accurate salivary gland duct geometry gives us confidence in parameters such as duct cell membrane area, volume, lumen length and radius. We implement a much finer lumen discretisation resolution (1 µm step size), which resolves the saliva flow gradient more precisely. We model the unstimulated and stimulated gland cases separately and found the steady-state solution for the unstimulated case and the dynamic response for the stimulated case. Based on the experimental findings of aquaporin, ENaC and CaCC on the apical membrane of the ID cells, we challenge the traditional view of the ID and argue that its role is to secrete water without absorbing much Na$^+$ / Cl$^-$ or secreting K$^+$.

For the 3D reconstruction, we devised our cell growing approach because we did not have an image stack in which both the cell membranes and the inner duct were clearly visible throughout the stack. We expect that the final cell reconstruction is physically realistic because our cell “growing” was guided by measured spatial statistics. Based on a finite number of microscopy observations, we assumed that no real-world cell has sharp edges and that the inner duct region is generally circular in cross section. It is possible that this is not always the case.

In the identification of the salivary ducts, there was some “smearing” of the features in the image stack. We attributed this to breathing induced motion in the live mouse subject. This distortion was smoothed out in our reconstruction by virtue of the assumption that the duct inner region is circular in cross section and relatively smooth along its length. The duct centreline is discretised into linear segments to perform the duct compartmentalisation described in Appendix . This could have been done against the Bezier curve representation of the duct centreline, but discretising the centreline into linear segments significantly simplifies our calculation while preserving reasonable precision.

The type of stimulation we study in this work is pilocarpine stimulation. Pilocarpine is a cholinergic agonist that mimics the action of parasympathetic nervous system neurotransmitters. The parasympathetic nervous system regulates eating and digestion. When analysing the unstimulated case, we run the duct model to temporal equilibrium, where both the saliva and the duct cells are in steady states. The temporal steady-state solution can represent the basal state of a salivary gland where it is not stimulated in between meals. The model takes 20,000 s to reach the steady state.

Since we are modelling the stimulation of saliva that occurs during eating, it is reasonable to keep the stimulation period to a few minutes. In this case, we are more interested in the dynamic response of the model and examine the model transient state at 400 s time point. At that time, the duct model has not yet reached a steady state because the cells take time to adjust to the change in the lumen, and water flow across cells is slow. As shown in Fig. 16, the duct cells slowly expand upon stimulation, and the effect reverses as stimulation is turned off.

Our model reproduces many physiological features of the salivary gland duct. The intracellular ion composition and pH are consistently maintained within physiological levels for all SD and ID cells. The membrane potentials are also realistic. The ID has a low transepithelial potential ($\sim$ 10 mV), whereas the SD epithelium is more polarised ($\sim$ 50 mV). It has been shown in experiments that the SD has a high transepithelial potential (Schneyer 1970). Even though there are no direct measurements of the ID, we do know that the adjacent acinar cells have a low tight junctional membrane potential ($\sim$ 13 mV (Martin et al. 1973)), and thus, the ID cells could have the similar property.

The steady-state cell volume of the SD and ID cells is consistent with that observed from the microscopic images, with SD cells larger than ID ones. From the temporal response of the increased flow rate under stimulation as shown in Fig. 16, we notice that duct cells expand when the luminal fluid becomes more similar to the primary saliva (higher Na$^+$ and Cl$^-$, lower K$^+$). It appears that a duct cell swells when it is transporting ions faster. One possible mechanism that explains this is that as more Na$^+$ enters the cell through apical membrane, cellular osmolarity increases which causes the cell to swell.

As shown in Fig. 9, the steady-state volume of the SD cells varies along the duct. They are larger near the ID and smaller at the distal end of duct. The phenomenon of enlarged ductal cells at the junction of ID and SD naturally shows up in the rat submandibular gland. The granular duct is located between the ID and SD in the rat submandibular gland. The granular duct cells are much larger than the other duct cells, and the observation is especially prominent in male rats (Catalán et al. 2010). It is a possibility that the granular duct cells are large due to their high ion transport efficiency.

Since our 3D model is based on the image stack of a part of a mouse submandibular gland, the SD in our model is shorter than a realistic SMG SD. The ion transporter parameters in the model are tuned so that the saliva ion composition reaches a spatial steady-state by the end of the SD available and that primary saliva is completely converted to final saliva. Therefore, ion transport in our model may be occurring faster than in the real gland. Mathematically, we control how fast saliva composition changes along duct by scaling the ion transporter parameters by a time factor. The primary saliva is converted to final saliva within a shorter duct length if ion transport occurs faster. Table 7 shows that the ion transporter parameters are scaled up by such a factor, which is chosen to fit the total duct length.

Following the 3D geometry of the duct mesh, we show that it is possible to simplify our duct model by grouping together all duct cells of one type into one compartment. The tree-like structure of ID is simplified to one straight tube surrounded by ID cells. The simple model still produces accurate final saliva output under both unstimulated and stimulated conditions. This shows that the final saliva composition is insensitive to the detailed duct structure and we can obtain accurate solutions with a model resolution as coarse as having only one compartment for each type of salivary gland duct.

The duct model has some limitations. The current model can only reproduce experiments of the mouse parotid gland under pilocarpine stimulation. In salivary glands, it appears that the primary saliva composition is generally consistent across animal, gland types, and stimulation types (Mangos et al. 1973a, b, c; Young et al. 1971). In each case, the primary saliva is near isotonic with high [Na$^+$] (140–150 mM), low [K$^+$] (5–15 mM), high [Cl$^-$] (80–130 mM) and moderate [HCO$_3^-$] (30–60 mM). However, the final saliva [K$^+$], [Cl$^-$] and [HCO$_3^-$] vary widely. For example, the salivary duct can sometimes secrete a large amount of HCO$_3^-$ (in rat parotid gland with isoproterenol stimulation) or hardly secrete any (in mouse parotid gland with pilocarpine stimulation). Parasympathetic stimulation generally induces a saliva flow rate of about 6–8 times higher than the sympathetic stimulation (e.g. isoproterenol). However, even considering similar flow rates, the final saliva composition from different stimulation types are very different (Young et al. 1971). This indicates different duct responses with different stimulation type, a feature that is not included in our model.

**Fig. 20:**
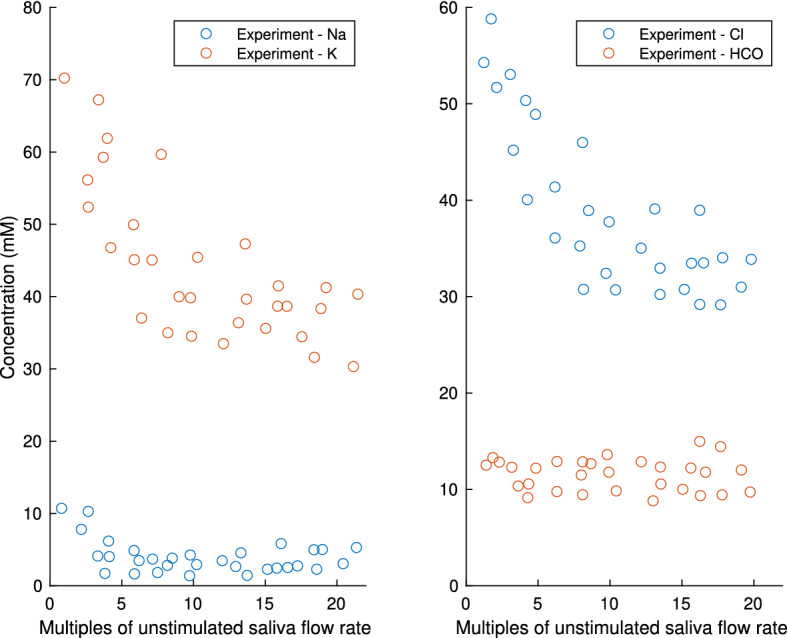
This plot shows the experimental dataset from Mangos et al. (1973c). It shows the mouse submandibular gland final saliva composition as a function of the saliva flow rates. This figure is to be compared with the parotid experimental data in Fig. 17 highlighting the differences in the trends of [Na$^+]_L$ and [Cl$^-]_L$. In the experiments, the saliva is collected for 3–10 min upon pilocarpine stimulation

The mouse submandibular gland final saliva measurement shows a different dependency on flow rate from the parotid gland. As shown in Fig. 20, the final [Cl$^-]_L$ is high at low flow rate but lower as flow rate increases. Since primary saliva [Cl$^-]_L$ is high, we deduce that in SMG, [Cl$^-]_L$ first decreases and then increases along the duct. We hypothesise that as the flow rate increases, the low [Cl$^-]_L$ saliva gets pushed down the duct more quickly, which is how the final saliva [Cl$^-]_L$ drops.

How can we achieve such a phenomenon in a duct model? We set up our parotid gland duct model such that all SD cells have the same ion transporter coefficients. Therefore, even though the ion flux magnitudes vary, flux directions are mostly uniform along the duct. As shown in Fig. 9, the luminal ion concentrations vary monotonically. To obtain first decreasing and then increasing [Cl$^-]_L$ in the SD in SMG, we propose varying transporter coefficients along the SD, so as to allow [Cl$^-]_L$ absorption at the proximal end of duct, then [Cl$^-]_L$ secretion towards the distal end of duct.

Given that our model can reproduce the ductal response of the mouse parotid gland with physiological cellular concentrations, pH and volume, we believe that justifies the overall structure of our model. To develop new models of other salivary glands, we only need to fit the ion transporter parameters to achieve different mode of ion secretion/absorption.

## Figures and Tables

**Fig. 21:**
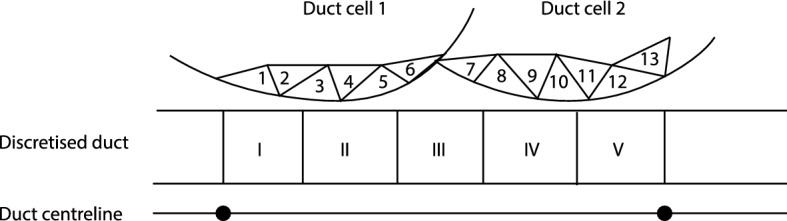
This figure shows the apical region mesh triangles of 2 duct cells against a section of the duct. The duct centreline is placed below the discretised duct to indicate that one duct segment is discretised into 5 discretisations. The triangle meshes are assigned to the duct discretisation where their centre of mass fall, and thus each triangle is only assigned to one discretisation, even though it may span two. Note that the duct centreline is depicted as a straight line, although in the reconstructed geometry, actual duct segments are jointed in angles

## Data Availability

The datasets generated and analysed during the current study are available from the corresponding author on reasonable request.

## Spatial Statistics

Table 5 shows the summary of the key dimensions measured from the image stack of the mouse SMG.

Based on the dimensions of the ID and SD cells, we estimate the volume of an ID cell to be approximately 1000 µm$^3$ (cube of 9.8 µm$^3$ sides), and SD cell to be 3000 µm$^3$ (wedge of 18 µm$^3$ sides). Since the nucleus occupies a large portion of the cell, the volume of intracellular fluid is estimated to be 200 µm$^3$ for ID cells and 1000 µm$^3$ for SD.

**Table 5:** Mouse submandibular gland microstructure statistics summary

	Diameter (µm)	SD	Count	SD	notes
Acinus					
Acinus	32.5	4.6			Spherical
Cells	16.9	3.3	13	1.5	Apical to basal, 3D wedge
Lumen	0.77	0.15			Cylindrical
Intercalated duct					
Duct - outer	23.2	3.3			Cylindrical
Duct - inner	1.6	0.25			Cylindrical
Cells	9.8	1.5			Apical to basal, cuboid
Cells in radial ring			5	0.5	
Striated duct					
Duct - outer	47.6	5.2			Cylindrical
Duct - inner	8	1.1			Cylindrical
Cells	18	3.1			Apical to basal, elongated cube wedge
Cells in radial ring			13	1	
Nuclei	4.2	0.59			Spherical

## Numerical Discretisation of Duct

The duct structure consists of a branching tree of line segments, as shown in Fig. 6. We discretise the duct by dividing each line segment into 1 µm lengths.

Since the duct cells exchange fluid and ions with the duct lumen, we need to establish how the cell mesh matches the duct discretisation. Figure 21 shows a section of the duct and the adjacent duct cell apical region mesh triangles. The duct segment is divided into 5 discretisations of 1 µm length. We assign a whole triangle to a duct discretisation if the centre of mass of the triangle falls within it. For example, triangle 1 and 2 is assigned to discretisation I, triangle 3–5 is assigned to II, and triangle 6 and 7 to III. This method determines which cell apical triangle exchanges molecules with duct discretisation.

## Mathematical Formulation of Ion Transporters

In this appendix, we include a brief summary of the mathematical equations in the model.

### NaK ATPase

We use the two-state model of Palk et al. (2010) to model the NaK ATPase pump rate. The two-state model is simplified from a four-state model (Smith and Crampin 2004) in which the rate parameters are fitted to. The pump flux $J_\mathrm{NKA}$, in units of moles per second, is given by

C1 $$\begin{aligned} J_\mathrm{NKA_e}=\alpha _\mathrm{NKA} r_\mathrm{NKA} \frac{[\mathrm{K}^+]_e^2 [\mathrm{Na}^+]_C^3 }{[\mathrm{K}^+]_e^2 + \beta _\mathrm{NKA} [\mathrm{Na}^+]_C^3 }, \end{aligned}$$

where $\beta _\mathrm{NKA}$ and $r_\mathrm{NKA}$ are the two parameters fitted to the four-state model, and $\alpha _\mathrm{NKA}$ is the transporter density coefficient. The *e* subscript, when used with ion concentrations, is a placeholder for one of the two extracellular compartments, i.e. $e = L$ indicates the luminal compartment and $e = I$ the interstitium. The *e* subscript, when used with ionic fluxes (*J*), is a placeholder for either apical or basolateral flux, i.e. $e = A$ indicates apical flux and $e = B$ basolateral.

It is usually assumed that NaK ATPases are localised on the basolateral membrane of the duct cells. However, weak staining of NaK ATPases on the apical membrane of SD cells has been observed through immunolabelling (unpublished data). Therefore, our model includes apical ATPases with a density lower than the basolateral one.

#### Ion Channels

Ion channels allow ions to passively diffuse under an electrochemical gradient which is quantified by the difference between membrane potential and the Nernst potential.

The Epithelial Na$^+$ Channel (ENaC) is shown to localise on the apical membrane of salivary gland duct cells (Catalán et al. 2010). It appears to be the major pathway of Na$^+$ reabsorption in the duct. The Nernst potential of Na$^+$ across the apical membrane ($V_A^\mathrm{Na}$) is given by

C2

To calculate the ion channel currents, we assume all the ion channels in the salivary gland duct cells have a linear I-V relationship (Catalán et al. 2010; Nakamoto et al. 2008). Therefore, the channel conductance can be represented as constant parameters. The current through the ENaC is the product of the channel conductance per area $G_\mathrm{ENaC}$, the electrochemical gradient ($V_A - V_A^\mathrm{Na}$) and the apical surface area $A_A$. Current has units of Ampere, and so dividing by Faraday’s Constant, *F*, gives the ion flux, $I_\mathrm{ENaC}$, as:

C3 $$\begin{aligned} I_\mathrm{ENaC} = \dfrac{A_A G_\mathrm{ENaC}}{F} (V_A - V_A^\mathrm{Na}) , \end{aligned}$$

in units of moles per second.

The Cystic Fibrosis Transmembrane conductance regulator (CFTR) is an anion channel that is present on the apical membrane of the duct cells. CFTR also demonstrates a limited permeability to HCO$_3^-$, with the permeability of Cl$^-$/HCO$_3^-$ shown to be roughly four-fold (Poulsen et al. 1994). Therefore, we take the HCO$_3^-$ conductance to be 0.25 times that of Cl$^-$. The Cl$^-$ and bicarbonate fluxes are given by

C4

The MaxiK channel is observed in the luminal membrane of the submandibular gland striated and excretory duct cells (Nakamoto et al. 2008). It has also been shown to be the major pathway of K$^+$ secretion in the salivary gland. MaxiK channels are activated by Ca$^{2+}$. The K$^+$ flux in units of moles per second is

C5

Fong et al. (2017) modelled the IK1 channel as the K$^+$ channel in the basolateral membrane; however, there seems to be no evidence of the presence of IK1 channel in the salivary gland duct cell, only the pancreatic duct cell (Lee et al. 2012). Nevertheless, our model shows that a basolateral K$^+$ conductance is required to control the basolateral membrane potential. ATP-sensitive K$^+$ channels have been detected in the basolateral membrane of rat submandibular gland duct cells (Zhou et al. 2010) and human submandibular gland duct cell line (Liu et al. 1999). Therefore, we include a basolateral K$^+$ current $I_{K_B}$ in the model, with the flux in units of moles per second as:

C6

#### Ion Cotransporters

The Na$^+$/H$^+$ exchangers, or NHEs, are a family of electroneutral ion cotransporters that extrude one proton in exchange for one Na$^+$. They serve to maintain the intracellular pH under varying intra- and extracellular HCO$_3^-$ concentrations. 3 isoforms of NHEs have been found in the salivary glands: NHE1, NHE2 and NHE3. NHE1 is localised in the basolateral membrane of duct cells, whereas NHE2 and NHE3 are found in the apical membrane (He et al. 1996; Park et al. 1999).

For the ion exchange rate, we adopt a two-state flux expression, introduced in the appendix and supplementary materials of Crampin and Smith (2006). The two-state model is a simplification of the six-state model of the transport cycle, with the assumption of rapid ion binding and unbinding to the exchanger. In this model, the exchanger concentration flux is determined by the ion concentrations to either sides of the membrane. The mole flux $J_\mathrm{NHE}$ is the product of concentration flux and cell volume $w_C$, as:

C7 $$\begin{aligned} J_{\mathrm{NHE}_e}=w_C \alpha _\mathrm{NHE} \frac{k_1^+ k_2^+ [\mathrm{Na}^+]_e [\mathrm{H}^+]_C - k_1^- k_2^- [\mathrm{Na}^+]_C [\mathrm{H}^+]_e}{k_1^+ [\mathrm{Na}^+]_e + k_2^+ [\mathrm{H}^+]_C + k_1^- [\mathrm{H}^+]_e + k_2^- [\mathrm{Na}^+]_C}. \end{aligned}$$

The *k*’s are the transition rate constants in the Na$^+$/H$^+$ exchange model, and are obtained from experimental data. $\alpha _\mathrm{NHE_A}$ is a dimensionless coefficient that represents the NHE density on the cell membrane. Since NHE activity in both the apical and basolateral membranes was shown to be similar (Zhao et al. 1995), we can use the same $\alpha$ coefficient for both membranes.

Slc26 transporters are responsible for anion transportation across cell membranes. Among the family of transporters, Slc26a6 and Slc26a4 are found in the salivary gland duct cells (Shcheynikov et al. 2008). Slc26a4 functions as an electroneutral Cl$^-$/I$^-$/HCO$^-$ exchanger and is expressed in the lumenal membrane (Shcheynikov et al. 2008). Slc26a6, on the other hand, functions as an electrogenic Cl$^-$/HCO$^-$ exchanger with a 2HCO$^-$/1Cl$^-$ stoichiometry (Ko et al. 2002). Both Slc26a6 and Slc26a4 are referred to as anion exchangers (AE). In this work, we do not differentiate between the two transporters and use an electroneutral model to quantify the flux of Cl$^-$/HCO$^-$ exchange. The flux expression is derived in the same way as the NHE exchanger (Crampin and Smith 2006), given by

C8 $$\begin{aligned} J_{\mathrm{AE}_e}=w_C\alpha _\mathrm{AE} \frac{k_3^+ k_4^+ [\mathrm{Cl}^-]_e [\mathrm{HCO}_3^-]_C - k_3^- k_4^- [\mathrm{Cl}^-]_C [\mathrm{HCO}_3^-]_e}{k_3^+ [\mathrm{Cl}^-]_e + k_4^+ [\mathrm{HCO}_3^-]_C + k_3^- [\mathrm{HCO}_3^-]_e + k_4^- [\mathrm{Cl}^-]_C}. \end{aligned}$$

Cl$^-$/HCO$^-$ activity has been detected in both the luminal and basolateral membrane of duct cells but shown to be more active in the luminal membrane (Zhao et al. 1995). Therefore, we use a higher rate coefficient $\alpha _\mathrm{AE2}$ for the luminal membrane to represent a denser expression of AE2.

The Na$^+$/HCO$_3^-$ cotransporters, NBC, have been shown to exist in the salivary duct gland. The activity of the electrogenic NBC1 subtype has been demonstrated in both the apical and basolateral membranes through immunolabelling and functional studies (Li et al. 2006). The NBC1 found has a stoichiometry of 1Na$^+$/2HCO$_3^-$ or 1Na$^+$/3HCO$_3^-$ (Romero and Boron 1999). Electroneutral NBC3 has been found in the apical membrane of the duct (Luo et al. 2001). As a simplification, we use one electroneutral equation to model both types of NBC. The flux $J_\mathrm{NBC}$ is derived in a similar way as the NHE exchanger, except with opposite ion movement directions (Crampin and Smith 2006), given by

C9 $$\begin{aligned} J_{\mathrm{NBC}_e}=w_C\alpha _\mathrm{NBC} \frac{k_5^+ k_6^+ [\mathrm{Na}^+]_C [\mathrm{HCO}_3^-]_C - k_5^- k_6^- [\mathrm{Na}^-]_e [\mathrm{HCO}_3^-]_e}{k_5^+ [\mathrm{Na}^+]_C [\mathrm{HCO}_3^-]_C +k_6^+ k_5^- + k_6^- [\mathrm{Na}^-]_e [\mathrm{HCO}_3^-]_e}. \end{aligned}$$

#### Paracellular Pathway

Ions also passively diffuse through the tight junctions. The transcellular membrane potential $V_T$ is the difference between apical and basolateral membrane potentials: $V_T = V_A - V_B$. Similar to the cell membrane, the transcellular Nernst potential depends on the luminal and interstitial ion concentration ratio. We assume that Na$^+$, K$^+$ and Cl$^-$ can pass through the tight junctions. The corresponding Nernst potentials, in unit of mV, are

C10

Assuming a linear I-V relationship, the rate of diffusion is set by a constant conductance coefficient. The paracellular currents are given by

C11 $$\begin{aligned} \begin{aligned} I_{P}^\mathrm{Na}&= \dfrac{A_A G_P^\mathrm{Na}}{F} (V_T - V_P^\mathrm{Na}), \\ I_{P}^\mathrm{K}&= \dfrac{A_A G_P^\mathrm{K}}{F} \left( V_T - V_P^\mathrm{K} \right) , \\ I_{P}^\mathrm{Cl}&= \dfrac{A_A G_P^\mathrm{Cl}}{F} (V_T - V_P^\mathrm{Cl}). \end{aligned} \end{aligned}$$

For the sign convention, a positive paracellular current is when positive charges travel from the interstitium to the lumen.

We argue that the salivary duct has leaky tight junctions at the proximal end, i.e. a high paracellular conductance/low resistance. This is because of the physical proximity of the ID with the acinus. The acinar cells have leaky tight junctions, as shown by the large paracellular Na$^+$ flux generated upon stimulation (Nauntofte 1992). It is reasonable to assume the two adjacent cell types share this characteristic to some degree. As saliva reaches the distal end of the duct, it becomes more hypotonic. To ensure saliva remains hypotonic, the duct epithelium has to be tight, i.e. having low paracellular conductance/high resistance. Therefore, the paracellular conductance of the ID will be greater than the SD.

#### Bicarbonate Buffering

CO$_2$ gas dissolves in and reacts with water molecules to produce carbonic acid which then hydrolyses into bicarbonate and proton. This reaction is key for pH buffering in both the cells and the saliva (Kivelä et al. 1999). In our model, we omit the intermediate step and assume the reaction

where the transition parameters $k_\mathrm{buf}^+$ and $k_\mathrm{buf}^-$ determine the reaction rates. The bicarbonate and proton generating flux, in the cellular and luminal compartment, are given by

C12

### Fluid Transport

We assume water flows across the cell membrane through aquaporins and that there is no water transport through the tight junctions between the ID cells. The flow rate is proportional to the osmolarity gradient across the membrane. The apical and basolateral water fluxes, in units of µm$^3$/s, are given by

C13 $$\begin{aligned} J_A&= A_A L_A V_W (\mathrm{OS}_L - \mathrm{OS}_C), \end{aligned}$$

C14 $$\begin{aligned} J_B&= A_B L_B V_W (\mathrm{S}_C - \mathrm{OS}_I), \end{aligned}$$

where $L_A$ and $L_B$ are the permeability coefficients of cell membrane, $A_A$ and $A_B$ are the apical and basolateral membrane areas and $V_W$ is the partial molar mass of water. $\mathrm {OS}_\dagger$ is the osmolarity of compartment $\dagger$, given by

C15 $$\begin{aligned} \begin{aligned} \mathrm{OS}_C&= [\mathrm{Na}^+]_C + [\mathrm{K}^+]_C + [\mathrm{Cl}^-]_C + [\mathrm{HCO}_3^-]_C + [\mathrm{CO}_2]_C + \frac{\chi _C}{w_C}, \\ OS_L&= [\mathrm{Na}^+]_L + [\mathrm{K}^+]_L + [\mathrm{Cl}^-]_L + [\mathrm{HCO}_3^-]_L + [\mathrm{CO}_2]_L + \phi _L, \\ \mathrm{OS}_I&= [\mathrm{Na}^+]_I + [\mathrm{K}^+]_I + [\mathrm{Cl}^-]_I + [\mathrm{HCO}_3^-]_I + [\mathrm{CO}_2]_I + \phi _I. \end{aligned} \end{aligned}$$

The cellular osmolarity equals the sum of cellular ion concentrations plus other protein molecules in the cell. $\chi _C$ represents the total moles of intracellular protein molecules. $\phi _L$ and $\phi _I$ represent the protein molar concentration in the luminal compartment and the interstitium.

Water movement towards the lumen is considered positive.

The water flow in the duct is governed by the ODE

C16 $$\begin{aligned} \frac{d V}{ d x} = J_W, \end{aligned}$$

where *V* is the volumetric flow rate of water, in units of µm$^3$/s, and $J_W$ is the water flux per unit length of duct, in units of µm$^2$/s. Note that $J_W$ is only nonzero in the ID.

We discretise the spatial dimension x and thus convert Eq. C16 to an algebraic equation

C17 $$\begin{aligned} \frac{V^{i-1} - V^i}{\nabla x} = J_W^i, \end{aligned}$$

where $J_W^i$ is the water flux per unit length in the *i*th duct segment and $V^{i-1}$ and $V^{i}$ is the volumetric inflow and outflow rate of the *i*th duct segment.

### Full System of ODEs

#### Cellular Compartment

The cellular variables are the membrane potentials, cell volume and cellular ion concentrations. The cell membrane is a thin electrical insulator that separates opposite charges and the membrane potentials are induced by currents across the membranes. Equations C18 and C19 define the membrane potentials, based on the capacitor relationship $C\dfrac{dV}{dt}=I$, where *C* is the capacitance, *V* is the voltage, and *I* is the current across the capacitor. Since the cell volume is a variable, we track the mole amount of the ions instead of the concentrations (Eqs. C21–C26).

C18 $$\begin{aligned}&\frac{C_A}{F} \frac{dV_A}{dt} = J_\mathrm{NKA_A} + I_\mathrm{ENaC} + I_\mathrm{BK} + I_\mathrm{CFTR} + I_\mathrm{CFTR_B} + I^P_\mathrm{Na} + I^P_\mathrm{K} + I^P_\mathrm{Cl}, \end{aligned}$$

C19 $$\begin{aligned}&\frac{C_B}{F} \frac{dV_B}{dt} = J_\mathrm{NKA_B} + I_\mathrm{K_B} - I^P_\mathrm{Na} - I^P_\mathrm{K} - I^P_\mathrm{Cl}, \end{aligned}$$

C20 $$\begin{aligned}&\frac{dw_C}{dt} = J_B - J_A, \end{aligned}$$

C21 $$\begin{aligned}&\frac{d([\mathrm{Na}^+]_C \cdot w_C)}{dt} = -I_\mathrm{ENaC} - 3(J_\mathrm{NKA_B}+J_\mathrm{NKA_A}) + J_\mathrm{NBC} + J_\mathrm{NHE_A} + J_\mathrm{NHE_B}, \end{aligned}$$

C22 $$\begin{aligned}&\frac{d([\mathrm{K}^+]_C \cdot w_C)}{dt} = -I_\mathrm{BK} - I_\mathrm{K_B} + 2(J_\mathrm{NKA_B}+J_\mathrm{NKA_A}), \end{aligned}$$

C23 $$\begin{aligned}&\frac{d([\mathrm{Cl}^-]_C \cdot w_C)}{dt} = I_\mathrm{CFTR} + J_\mathrm{AE_A} + J_\mathrm{AE_B}, \end{aligned}$$

C24 $$\begin{aligned}&\frac{d([{\mathrm{HCO}_3}^+]_C \cdot w_C)}{dt} = I_\mathrm{CFTR_B} + J_\mathrm{NBC} - J_\mathrm{AE_A} - J_\mathrm{AE_B} + J_{\mathrm{buf}_C}, \end{aligned}$$

C25 $$\begin{aligned}&\frac{d([\mathrm{H}^+]_C \cdot w_C)}{dt} = - J_\mathrm{NHE_A} - J_\mathrm{NHE_B} + J_{\mathrm{buf}_C}, \end{aligned}$$

C26 $$\begin{aligned}&\frac{d([\mathrm{CO}_2]_C \cdot w_C)}{dt} = - J_{\mathrm{CDF}_A} - J_{\mathrm{CDF}_B} - J_{\mathrm{buf}_C}. \end{aligned}$$

#### Luminal Compartment

For each lumen segment, we track the cellular concentrations based on the ion transport rates and the flux of ion due to the bulk movement of saliva flow. The system equations are

C27 $$\begin{aligned}&\frac{d[\mathrm{Na}^+]_L}{dt} = v_\mathrm{in}[\mathrm{Na}^+]_P - v_\mathrm{out} [\mathrm{Na}^+]_L + \frac{1}{w_L}(I_\mathrm{ENaC} + 3J_\mathrm{NKA_A} \!+\! J_\mathrm{NBC} \!+\! J_\mathrm{NHE_A}), \end{aligned}$$

C28 $$\begin{aligned}&\frac{d[\mathrm{K}^+]_L}{dt} = v_\mathrm{in}[\mathrm{K}^+]_P - v_\mathrm{out} [\mathrm{K}^+]_L + \frac{1}{w_L}(I_\mathrm{BK} + I_\mathrm{K_B} - 2J_\mathrm{NKA_A}), \end{aligned}$$

C29 $$\begin{aligned}&\frac{d[\mathrm{Cl}^-]_L}{dt} = v_\mathrm{in}[\mathrm{Cl}^-]_P - v_\mathrm{out} [\mathrm{Cl}^-]_L + \frac{1}{w_L}( - I_\mathrm{CFTR} - J_\mathrm{AE_A}), \end{aligned}$$

C30 $$\begin{aligned}&\frac{d[{\mathrm{HCO}_3}^-]_L}{dt} = v_\mathrm{in}[\mathrm{HCO}^-]_P - v_\mathrm{out} [\mathrm{HCO}_3^-]_L\nonumber \\&\quad + \frac{1}{w_L}(- I_\mathrm{CFTR_B} - J_\mathrm{NBC} + J_\mathrm{AE_A} + J_{\mathrm{buf}_A}), \end{aligned}$$

C31 $$\begin{aligned}&\frac{d[\mathrm{H}^+]_L}{dt} = v_\mathrm{in}[\mathrm{H}^+]_P - v_\mathrm{out} [\mathrm{H}^+]_L + \frac{1}{w_L}(J_\mathrm{NHE_A} + J_{\mathrm{buf}_A}), \end{aligned}$$

C32 $$\begin{aligned}&\frac{d[\mathrm{CO}_2]_L}{dt} = v_\mathrm{in}[\mathrm{CO}_2]_P - v_\mathrm{out} [\mathrm{CO}_2]_L + \frac{1}{w_L}(J_{\mathrm{CDF}_A} - J_{\mathrm{buf}_A}), \end{aligned}$$

where $v_\mathrm{in}$ and $v_\mathrm{out}$ are volumetric inflow and outflow rates for each lumen segment and $v_\mathrm{out}$ = $v_\mathrm{in} + J_A$. $[\mathrm{N}]_P$ is the upstream saliva concentration of ion N. For the first lumen segment, $[\mathrm{N}]_P$ is the primary saliva concentration.

## Model Parameters

Table 6 contains the values of the physical constants and the intracellular concentrations used in the model.

Table 7 lists the density coefficients of the ion channels and cotransporters that have been fitted to match the experimental saliva measurements. The densities for the ID cells are different from the SD cells.

Tables 8 and 9 contain the rate constants in the model expression for the Na-K-ATPase and the acid transporters, which are all from previous work of model fitting and simplification.

**Table 6:** Table of the physical parameter values used in the model

Parameters	Value	Unit	Description
*R*	8.13144621	J mol$^{-1}$ K$^{-1}$	Ideal gas constant
*T*	310	K	Body Temperature
*F*	96485.3329	C mol$^{-1}$	Faraday constant
$V_w$	18e12	µm$^{3}$ mol$^{-1}$	Partial molar volume of water
$w_C^o$	1000	µm$^{3}$	Initial cell volume
$\chi _C$	4e $-$ 14	moles	Moles of intracellular protein ($\dfrac{\chi _C}{w_C^o} = 40$ mM)
$z_{\chi }$	$-$ 1.5		Valence of intracellular protein
$\phi _L$	0.2	mM	Concentration of digestive enzymes
$\phi _I$	10.92	mM	Concentration of interstitial solute

**Table 7:** The ion channel and transporter density coefficients listed for the ID and the SD. All parameters (except $L_A$ and $L_B$) are scaled up by 40, so that the final saliva reaches spatial steady states at the duct end

Notation	Intercalated	Striated	Unit	Description
$G_\mathrm{ENaC}$	0.01	0.8	S m$^{-2}$	Apical ENaC channel
$G_\mathrm{CaCC}$	1	0	S m$^{-2}$	Apical CaCC channel
$G_\mathrm{CFTR}$	0	15	S m$^{-2}$	Apical CFTR channel
$G_\mathrm{BK}$	0.1	1	S m$^{-2}$	Apical Maxi-K channel
$G_{K_B}$	0.1	4	S m$^{-2}$	Basolateral K$^+$ channel
$\alpha _{\mathrm{NKA}_B}$	0.1E $-$ 8	0.8E-8	mol m$^{-2}$	Basolateral NaK ATPase
$\alpha _{\mathrm{AE2}_A}$	0.002	0.002	–	Apical anion exchanger
$\alpha _{\mathrm{AE2}_B}$	0.005	0.005	–	Basolateral anion exchanger
$\alpha _{\mathrm{NHE}_A}$	0.0002	0.0002	–	Apical Na$^+$/H$^+$ exchanger
$\alpha _{\mathrm{NHE}_B}$	0.0005	0.0005	–	Basolateral Na$^+$/H$^+$ exchanger
$\alpha _{\mathrm{NBC}_A}$	0	1500	–	Apical Na$^+$/HCO$_3^-$ cotransporter
$\alpha _{\mathrm{NBC}_B}$	2253	2253	–	Basolateral Na$^+$/HCO$_3^-$ cotransporter
$G_\mathrm{Na}^P$	0.3	0.15	S m$^{-2}$	Paracellular Na$^+$ conductance
$G_{K}^P$	0.3	0.3	S m$^{-2}$	Paracellular K$^+$ conductance
$G_\mathrm{Cl}^P$	0.5	0.35	S m$^{-2}$	Paracellular Cl$^-$ conductance
$L_A$	0.6	0	µm/s	Apical water permeability
$L_B$	0.6	0.6	µm/s	Basolateral water permeability

**Table 8:** Ion transporter constant coefficients for the Na-K-ATPase and the bicarbonate buffering reaction

Parameters	Value	Unit
Na-K-ATPase		
$r_\mathrm{NKA}$	1.305e $-$ 3	mM$^{-3}$s$^{-1}$
$\beta _\mathrm{NKA}$	0.647e $-$ 4	mM$^{-1}$
HCO$_3^-$ buffer		
$k^+_\mathrm{buf}$	0.03	s$^{-1}$
$k^-_\mathrm{buf}$	20	mM$^{-1}$s$^{-1}$
$P_{\mathrm{CO}_2}$	50	s$^{-1}$

**Table 9:** Table of the fitted parameters in the simplified models of the acid transporter rates

	k$^+$	k$^-$
NHE		
1	1.4159 $\times$ 10$^3$	1.4284 $\times$ 10$^{11}$
2	2.5296 $\times$ 10$^9$	1.7857 $\times$ 10$^2$
AE		
3	5.8599	1.0652 $\times$ 10$^8$
4	9.3124 $\times$ 10$^7$	5.1418
NBC		
5	$-$ 6.039 $\times$ 10$^{-1}$	1.0 $\times$ 10$^8$
6	1.0004 $\times$ 10$^8$	$-$ 1.9352 $\times$ 10$^{-1}$
